# Metagenomic polymorphic toxin effector and immunity profiling predicts microbiome development and disease-related dysbiosis

**DOI:** 10.1128/msystems.00305-26

**Published:** 2026-05-22

**Authors:** Hunter W. Schroer, Francesco Beghini, Juan Antonio Raygoza Garay, Nicholas A. Christakis, Dustin E. Bosch

**Affiliations:** 1 Civil, Architectural & Environmental Engineering, Missouri University of Science and Technology 14717, Rolla, Missouri, USA; 2 Yale Institute for Network Science, Yale University 5755, New Haven, Connecticut, USA; 3 Department of Communication Sciences & Disorders, University of Iowa 33774 , Iowa City, Iowa, USA; 4 Holden Comprehensive Cancer Center, Iowa City, lowa, USA; 5 Departments of Statistics and Data Science, Biomedical Engineering, and Medicine, Yale University 5755, New Haven, Connecticut, USA; 6 Pathology Department, The University of Iowa 4083, Iowa City, Iowa, USA; The University of Hong Kong, Hong Kong, Hong Kong

**Keywords:** toxin secretion system, machine learning, non-genetic inheritance, gut microbial ecology, vertical transmission

## Abstract

Bacteria use antagonistic interbacterial weapons, such as polymorphic toxin secretion systems (TSS), to compete for niches in the human gut microbiome. We hypothesized that TSS influence gut microbiome development and disease-related dysbiosis. We developed a bioinformatic marker gene approach (PolyProf) to quantify TSS including ~200 effector and immunity genes and applied it to ~15,000 publicly available human metagenomes. PolyProf alpha and beta diversity readily distinguished 12 different human disease states and enabled the construction of highly accurate linear regression classifier machine learning models. Elastic net machine learning models integrating bacterial taxonomy with PolyProf had strong predictive value for 12 disease states, outperforming models utilizing taxonomy alone. During microbiome development in the first year of life, PolyProf alpha diversity increases, and beta diversity becomes increasingly like the maternal microbiome, influenced by vertical transfer, delivery mode, and breastfeeding. PolyProf is related to strain sharing among adults through social interactions. In summary, TSS genes strongly correlate with microbiome development and interpersonal strain sharing, suggesting roles for interbacterial antagonism. Since PolyProf distinguishes diverse adult disease statuses, these dynamics may contribute to non-genetic inheritance.

**IMPORTANCE:**

Previous research has demonstrated that bacteria compete within the gut microbiome using toxin secretion systems (TSS). How TSS contribute to human microbiome development and the microbiome alterations observed in human diseases is not known. This study develops a new bioinformatic tool for profiling TSS-related genes in metagenomic data. Application of this approach to large-scale human fecal metagenomic data demonstrates the dynamic association of TSS during microbiome development, including the exchange of strains among social contacts. TSS gene abundance patterns are highly predictive of 12 disease states. This study advances the field by enabling TSS profiling in metagenomes and by identifying disease and microbiome development biomarkers that provide hypotheses for future mechanistic studies and may be useful for disease diagnosis.

## INTRODUCTION

Many human diseases significantly alter the intestinal ecosystem, which naturally results in changes to bacterial communities away from the “healthy” state, referred to as dysbiosis (1). Although dysbiosis is often an effect of disease, causal links for the dysbiotic microbiome in the pathogenesis and progression of several diseases, such as inflammatory bowel disease (IBD) and obesity, have also been established (1, 2). Mechanisms include bacterial community modulation of host immunity and production of disease-promoting proteins and metabolites (3–5). Bacteria use a complex arsenal of toxins to compete for niches in ecosystems with limited resources like the human gut, which we hypothesize could be a driver in microbiome development and dysbiosis (6, 7).

Development of the gut microbiome in infancy is a highly dynamic process that involves the acquisition of bacteria from the environment and rapidly changing host factors, such as immune system development (8–11). In the case of maternal primary caregiving and/or breastfeeding, there is a tendency for the infant gut microbiome to become increasingly like the maternal gut microbiome (8, 11). Vertical strain transfer has been demonstrated in several studies to occur at birth and throughout the first year of life through nutritional sources and close contact (12–16). In addition, infant microbiome development is influenced rather dramatically by the delivery mode (vaginal delivery [SVD] vs Cesarean section [CSD]). Since dysbiosis is a prominent feature in many diseases and dysbiotic microbiomes play causal roles in several diseases, it is distinctly possible that vertical transmission of gut microbiome components may underlie non-genetic disease inheritance patterns (17, 18).

Polymorphic toxin effectors utilized in interbacterial antagonism compromise central processes required for cell survival, such as the maintenance of membrane, cell wall, and genome integrity (6). Most effectors are highly efficient enzymes, such that delivery of one or a few effector proteins is fatal to a non-immune recipient bacterium. In the interbacterial antagonism arms race, polymorphic effector genes have proliferated, giving rise to sequence diversity and effector fusions with secretion-related domains like VgrG, PAAR, and LXG (7, 19). Because of high sequence diversity, frequent horizontal transfer, and technical challenges in molecular studies of toxic proteins, bioinformatic identification and function prediction of polymorphic toxin effectors are difficult (7, 20, 21). Effector-encoding cells protect themselves and kin from intoxication with immunity proteins, often encoded immediately 3′ on the genome from the effector gene. Immunity can be acquired by inheritance or horizontal transfer of toxin secretion systems, many of which are transferable via plasmids or other mobile genetic elements (22–24). Another defensive strategy is the acquisition of “orphan” immunity genes without the corresponding effector or energetically costly secretion system (22, 25). For example, Bacteroidales use genetically mobile arrays of immunity genes or acquired interbacterial defense systems (AID) to confer protection from several effector classes (25).

Polymorphic toxin effectors are delivered to recipient competitors through several conserved mechanisms (6). For example, colicins are synthesized and released from dying *E. coli* and co-opt membrane transporters to enter and attack recipient bacteria (6). The contact-dependent growth inhibition (CDI), or type V secretion system (T5SS), delivers effectors to contacting cells through cell surface-receptor interactions (6). The type VI secretion system (T6SS) forms an apparatus structurally related to bacteriophage, comprising TssA-R proteins (6, 26). A contractile sheath propels a tip structure decorated with an effector(s) to deliver effectors into the recipient periplasm and/or cytoplasm (27). T6SSs are divided into genetically related clades with different taxonomic distributions, and T6SS^iii^ is exclusively distributed among Bacteroidales (28). T6SS^iii^ membrane complexes are composed of TssQ and other structural proteins that are lacking in the T6SS found in Proteobacteria (29). Esx, or type VII secretion systems (T7SS), were initially described in Mycobacteria, and homologs are widespread in Bacillota (30). EssB is one of several conserved integral membrane proteins required for secretion (6).

T6SS^iii^ are known to mediate interbacterial antagonism and selective colonization in the human gut microbiome (25, 28, 31). Inactivation of the T6SS in Bacteroidales impairs competitive growth *in vitro* and competitive colonization of gnotobiotic mice (25, 28, 31). A human gut metagenome analysis of T6SS^iii^ and associated effector/immunity genes demonstrated that the GA3 architecture systems found in *Bacteroides fragilis* are associated with increased *Bacteroides* abundance and are enriched in infant microbiomes (32). Dominant effector types are proposed to competitively exclude colonization by related, but non-immune Bacteroidales (24, 25, 32, 33), potentially contributing to individual microbiome stability. The relationship between polymorphic toxin secretion systems in the gut microbiome and human diseases remains an open area of research. One major barrier to identifying disease-relevant gut microbiome effector/immunity genes is the lack of available tools for measuring their abundances in metagenomic data. We previously constructed hidden Markov models (HMMs) of some effectors and applied them to translated metagenomic sequencing reads (22). However, the approach relied upon sequences derived primarily from pathogens and environmental bacteria and adapted a bioinformatic approach that was not designed for metagenomic data analysis. Curated effector/immunity databases (7, 20, 21) are an excellent resource for sequence data but are similarly biased toward environmental bacteria and pathogens due to available genome sequences and historical research foci of secretion systems. Bioinformatic tools are not readily available for their application in microbiome research.

The prior research provides proof of concept that polymorphic toxin secretion genes may be disease-specific markers of dysbiosis. The model of competitive exclusion of non-immune Bacteroidales strains (32) and the prominence of this order in the effects of the delivery mode on infant microbiomes also suggests that polymorphic toxins may be an important component of gut microbiome development (9, 11, 32). We hypothesized that polymorphic toxin secretion system gene abundances change during infant microbiome development and are biomarkers of disease-related dysbiosis. The primary goals of this study are to i) develop a bioinformatics pipeline (PolyProf) for quantification of polymorphic toxin secretion genes in metagenomes, ii) describe the landscape of PolyProf in microbiome development and disease dysbioses, and iii) test the predictive value of PolyProf biomarkers for human disease diagnosis.

## RESULTS

### Development of a metagenomic polymorphic toxin effector/immunity profiling pipeline (PolyProf)

To identify disease associations with intestinal microbiome effectors and immunity genes, we constructed a marker protein sequence database. Effector, immunity, and select TSS genes were collected from 200,000 gut metagenome-assembled genomes (MAGs) (Mgnify) using sequence similarity searches with BLAST and HMMer (34–36). We restricted the initial database to the effector and immunity classes described by Zhang et al. (7) (Table S1; Fig. S1). The number of MAG-derived sequences in each class is indicated in Table S1 (median 105; quartile range 23–526). To estimate the degree of sequence variation for each marker, Shannon entropy was calculated from an alignment of each marker class (Table S1; mean 1.1, 95% C.I. 1.0–1.2). As for all metagenomic marker databases, PolyProf has a bias toward available genome sequences and likely does not capture the full sequence diversity of each effector, immunity, and TSS gene class. This customized marker gene database, made up of 181 gene markers, was used with HUMAnN (37) to quantify each effector, immunity, and TSS gene class in shotgun metagenomic data. We next tested the validity and performance characteristics of this tool, which we named PolyProf.

Because many TSS genes co-occur in bacterial genomes, co-abundance patterns are expected in metagenomic profiles. A high positive correlation of T6SS^iii^ marker abundances was observed across all data sets (Fig. S2), reflecting the expected co-abundance of 13 targets required for functional apparatus assembly in Bacteroidales. PolyProf does not distinguish sequences from the three T6SS^iii^ genetic architectures (GA1–3). Effector-immunity pairs are also expected to co-occur in metagenomes, as observed for Tde/Tdi (Fig. S2). However, strong linear effector/immunity co-abundance is not expected due to the preponderance of orphan immunity in the gut microbiome (25). Conversely, co-abundance of markers not related to direct effector/immunity interactions may arise, for example, due to genetic mobility of secretion systems and immunity gene arrays (AIDs) (24, 25, 33).

Marker gene quantitation can be affected by high sequence diversity within the target gene class or off-target detection due to sequence similarity with non-TSS genes. PolyProf database performance was assessed using simulated metagenomic data (38), generated from randomly selected MAGs with and without the corresponding effector/immunity genes (Fig. S3). As expected, most (175 out of 181) PolyProf marker abundance measurements correlated linearly with the relative abundance of the encoding MAGs. Exceptions were Ntox11 markers, which tended to underestimate (low sensitivity) true gene abundance, and TssR, Ntox30, LDpeptidase, and ToxREase4, which systematically overestimated abundance (low specificity). The markers with lower specificity exhibited off-target hits in marker-negative MAG simulation data but retained a positive linear correlation with marker-positive MAG abundances (Table S1). We compared PolyProf’s performance on simulated data to effector/immunity detection by HMMER with HMMs constructed from Zhang et al.’s effector/immunity sequences (7, 34). PolyProf had fewer off-target hits (better specificity), a better linear correlation with marker-positive MAG abundances, and equivalent limits of detection (sensitivity) compared to the HMM approach (Fig. S3; Table S1). We also assessed cross-mapping artifacts between PolyProf database sequences and a random selection of 100 MAGs negative for each marker using CROSSMAPPER (39). All markers had <0.5% cross-mapping (Table S1; median 0.006%, tertile 0.02%). We conclude that most PolyProf markers have good sensitivity and specificity in simulated metagenomic data.

We considered that PolyProf is likely influenced by taxonomy because some effector/immunity and secretion system genes are restricted to specific taxonomic groups. To quantify and visualize taxonomic distributions of each marker using gut MAGs (MGnify), we generated trees and calculated enrichment *Z*-scores by taxonomic group (available online at PolyProf GitHub site). We then used 1,537 metagenomes from the IBD cohorts, including many healthy controls, to calculate both PolyProf and taxonomic relative abundances (37). The contribution of taxonomy to PolyProf diversity was assessed by linear regression fitting on plots of the dominant NMDS from PolyProf vs the dominant NMDS from taxonomy beta diversity (Fig. S4). An R^2^ value of 0.11 can be interpreted as approximately 11% of the variance in PolyProf being explained by taxonomy. Partial redundancy analysis to evaluate the effect of taxonomy on PolyProf marker abundances in the entire data set identified a total constrained inertia of 12%. The findings indicate that a small but significant fraction of PolyProf variance is due to the differential abundance of bacterial taxa that tend to encode specific effector/immunity and secretion system genes. A small subset of the markers drives the influence of taxonomy on PolyProf. For example, exclusion of the Bacteroidales-restricted T6SS^iii^ gene markers decreases the taxonomy-explained PolyProf variance from ~11% to ~7%. In biological terms, the low correlation between taxonomy and PolyProf reflects other factors contributing to effector/immunity abundances, such as the selection for advantageous antagonism systems and horizontal transfer among diverse taxa. Because PolyProf and taxonomic beta diversity are not strongly correlated, they may have different and complementary predictive value in disease.

### Meta-analysis of large shotgun metagenomic gut microbiome data sets

**Fig 1:**
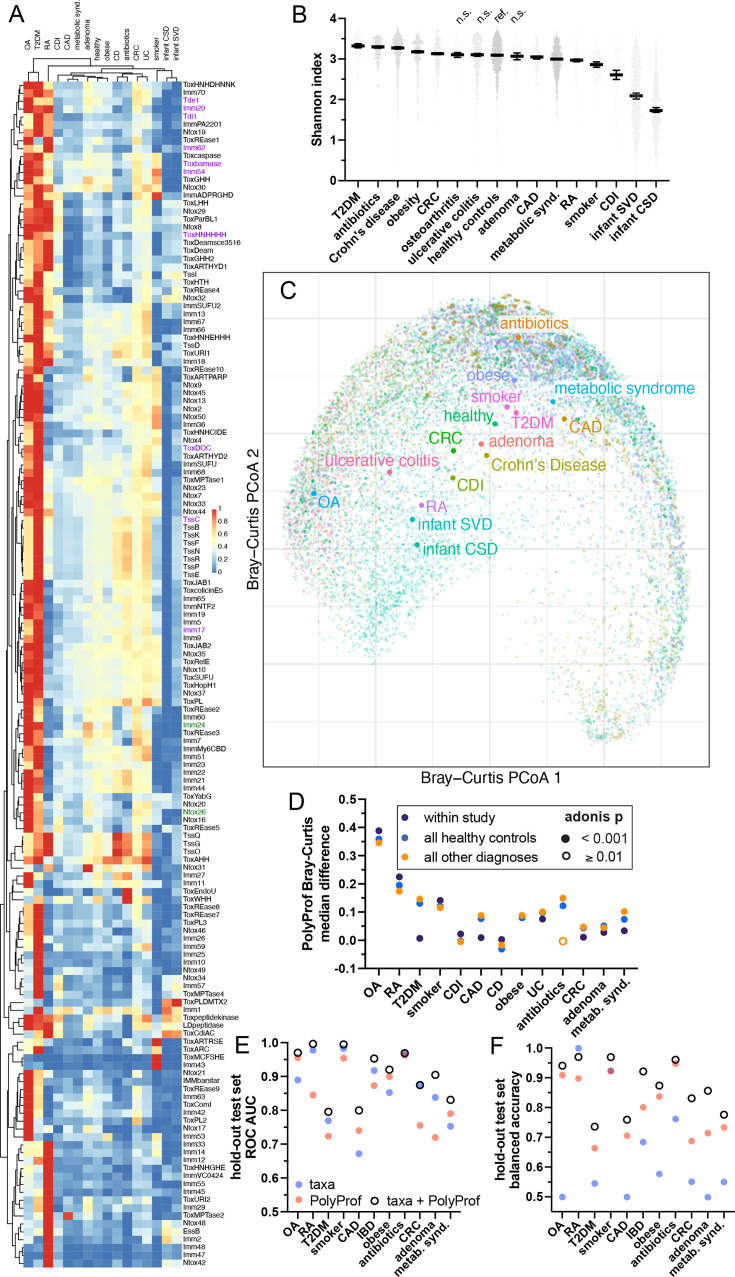
Gut metagenome polymorphic effector/immunity profiles distinguish disease states. (**A**) Marker gene abundances, centered log-ratio-transformed, are represented in a heat map with clustering by similarity. Osteoarthritis and type 2 diabetes (T2DM) subjects have the highest overall abundance of many effector/immunity classes and T6SS^iii^. Independently diagnosis-distinguishing markers identified by logistic regression are colored green for *P* < 0.001 and purple for *P* < 0.01. (**B**) Marker gene alpha diversity differs from “healthy control” comparators in all disease classes (adjusted *t*-test *P* < 0.01) other than colon adenoma, ulcerative colitis, and osteoarthritis. (**C**) Beta diversity also differed by diagnosis measured as Bray-Curtis dissimilarity principal coordinates analysis (PCoA). (**D**) Beta diversity differences held (adonis2 *P* < 0.001) in a 3-tier analysis for all diagnoses except the antibiotics-treated group. (**E and F**) Elastic net machine learning models were constructed to predict disease based on PolyProf, curated effectors, taxonomy, or combined taxonomy and PolyProf. Performance is measured on a 20% hold-out test set with receiver operating curve (ROC) and balanced accuracy. Abbreviations: OA, osteoarthritis; RA, rheumatoid arthritis; CSD, Cesarean delivery; SVD, vaginal delivery; CRC, colorectal carcinoma; CD, Crohn’s disease; UC, ulcerative colitis; CDI, *C. difficile* infection; met synd., metabolic syndrome.

We sought to identify microbiome effector/immunity diversity and abundance patterns that are associated with human disease. We applied PolyProf to ~15,000 human fecal metagenomes grouped by one of 16 diagnoses (Fig. S1; Table S2). A 3-fold analysis was then undertaken: (i) by diagnosis within a study, (ii) each diagnosis compared to pooled adult “healthy controls” across studies, and (iii) each diagnosis compared to all other metagenomes. The rationale for this approach was targeting effector/immunity profiles that are generalizable to populations with diverse health statuses. Global diversity of effector/immunity profiles exhibited significant differences by diagnosis (Fig. 1). Some individual markers, including the DNAse effector/immunity pair Tde/Tdi known to confer competitive advantages for Bacteroidales (22), were independently predictive of disease state using multivariate logistic regression (Fig. 1A). To determine how taxonomy may influence PolyProf disease associations, we applied multivariable linear modeling (MaAsLin), with and without adjustment for Bacteroidales abundance (Fig. S4). The relatively low T6SS^iii^ markers in infant microbiomes are influenced by Bacteroidales abundance, but their associations remain significant after adjustment for Bacteroidales. Other T6SS^iii^ disease associations, such as depletion in obesity and enrichment in ulcerative colitis, are not strongly affected by taxonomy (Fig. S4).

Marker gene alpha diversity (Shannon index) was either higher or lower than the “healthy controls” referent in all diagnostic groups except colorectal adenoma, ulcerative colitis (UC), and osteoarthritis (OA) (Fig. 1B). The highest PolyProf alpha diversity is observed in type 2 diabetes (T2DM) metagenomes. Much lower median profile alpha diversity was observed in infant microbiomes than in adults, but with high interindividual variability. This finding raises hypotheses that polymorphic toxins may play a role in microbiome development, building diversity from infancy to adulthood. Effector/immunity profile beta diversity also readily distinguished microbiomes by disease state (Bray-Curtis adonis2, *P* < 0.001) (Fig. 1C).

### Polymorphic toxin effector/immunity profiles predict disease states

The diversity patterns within the complete meta-analysis data set suggested that effector/immunity profiles may be predictive of disease state. However, differences in study design and methodology may also contribute to variance. To address this possibility, we compared beta diversity by diagnosis within each study (defined in Table S2) and compared it to the pooled healthy controls from the entire data set (Fig. 1D). Finally, we validated the correlation between PolyProf diversity and disease state using an independent cohort of village residents (Fig. S5) (40). The findings indicate that human diseases have distinct effector/immunity patterns that can be identified within a large population of subjects of diverse health statuses.

We next leveraged the large data set to generate machine learning predictors for each diagnosis, compared to all healthy controls and to all other diagnoses (Fig. 1E and F; Fig. S6 and S7). Using patient-grouped, 10-fold cross-validation, we optimized the elastic net parameters on the IBD cohort and tested the model on a 20% hold-out data set. Polymorphic effector/immunity profiles were predictive of IBD (hold-out receiver operator area under the curve [AUC] 0.87, balanced accuracy of 0.81 vs all other diagnoses, Fig. 1E and F), with predictive model performance meeting or exceeding current models built using taxonomy (41). Predictive models built with the same parameters were also highly predictive for the other data set diagnoses. Performance correlated with the training data set size (Fig. 1E and F; Fig. S6 and S7). Particularly high-performing models (testing area under the receiver operating curve [ROC] and balanced accuracy ≥ 0.85) were identified for osteoarthritis, smoking, IBD, obesity, and antibiotic use. Taken together, the polymorphic toxin effector/immunity profiles show strong potential for predicting disease-specific dysbiosis.

Because PolyProf and taxonomy are not highly interdependent, we reasoned that combined microbiome relative abundance and PolyProf data would likely enhance model performance. We generated elastic net models with taxonomy only or combined taxonomy and PolyProf (Fig. 1E and F; Fig. S6 and S7) to distinguish each individual diagnosis from all others. Taxonomy-only models tended to have comparable ROCs to PolyProf, but lower balanced accuracy (several models near 0.5, Fig. 1F). Combined taxonomy and PolyProf model performances on hold-out validation data sets were higher than those of models with either single data type for all diagnoses except rheumatoid arthritis, where all models were highly predictive. The additive effects of PolyProf and taxonomy on model performance were confirmed with Friedman tests (Fig. S6). The PolyProf elastic net diagnosis classifiers were further validated using independent, curated metagenomic data sets for CAD, IBD, obesity, CRC, and T2DM, obtained from ExperimentHub (42). As expected, the performance was lower on independent data, but all AUC and balanced accuracies were >0.5 (Fig. S7). Model feature stability over folds is also shown in Fig. S7.

### Infant delivery mode correlates with the toxin secretion system and effector/immunity profiles

Cesarean section delivery (CSD) markedly affects the intestinal microbiome composition of infants during the first year of life (10–12, 43). However, at 1 year and beyond, microbiomes of CSD and vaginally delivered (SVD) babies tend to converge and resemble the maternal microbiome in terms of species diversity (9, 11, 44). We hypothesized that polymorphic toxin secretion systems may be particularly active in the developing microbiome, modulating colonization by new strains through interbacterial antagonism. We tested this hypothesis by looking for PolyProf profile changes over time, stratified by the birth delivery mode.

**Fig 2:**
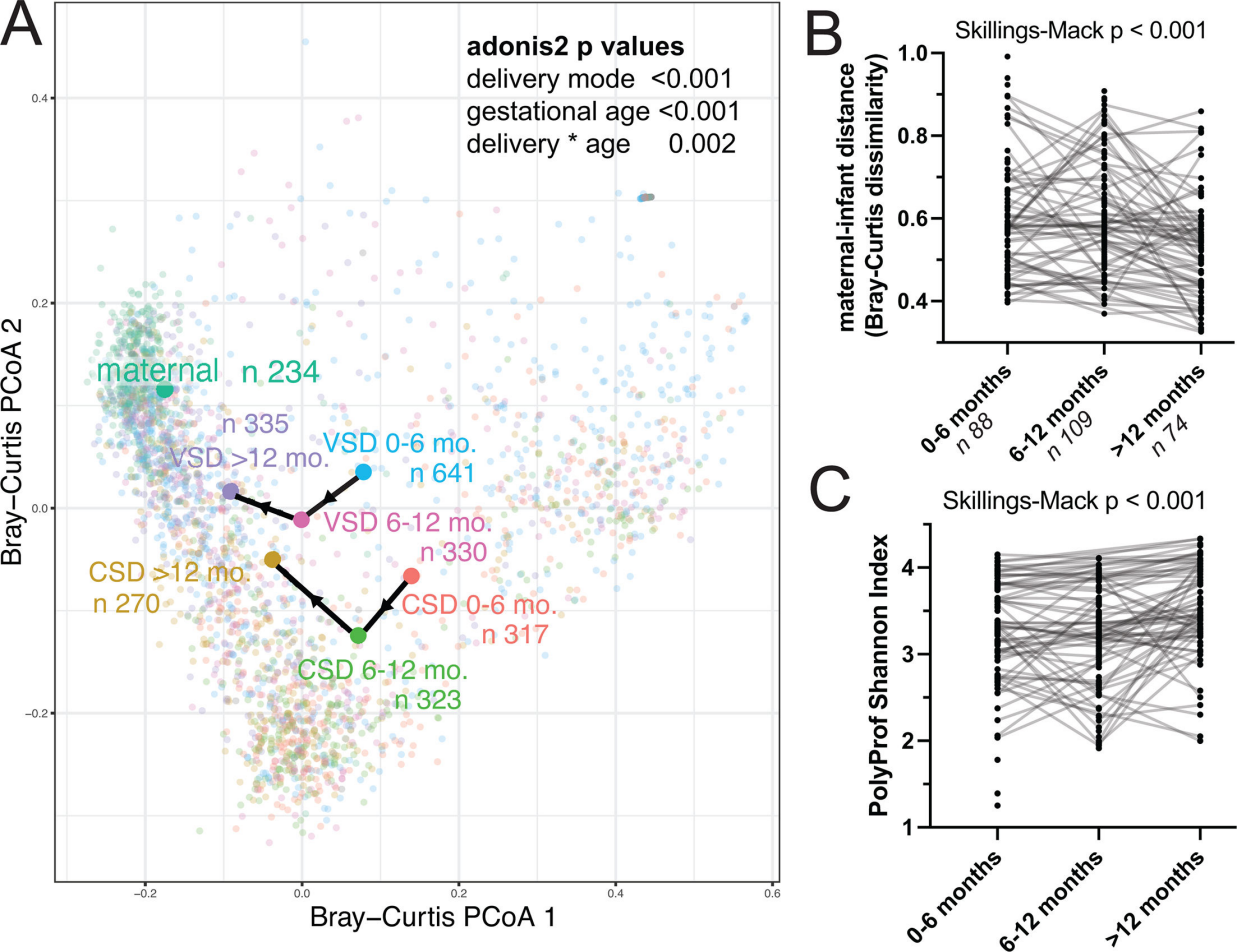
Infant microbiome polymorphic toxin effector/immunity profiles develop toward maternal profiles in the first year of life. (**A**) PolyProf beta diversity of infant microbiomes is distinct from maternal microbiomes. Infant PolyProfs are significantly affected by the delivery mode (VSD: vaginal delivery or CSD: Cesarean section). As infants age, PolyProf becomes more similar to maternal microbiomes, shown by centroids (large circles) and time course with black lines. For subjects with known maternal-infant pairings (connected with lines), the trend toward infant similarity with the maternal PolyProf over time is reflected as decreasing Bray-Curtis dissimilarity between maternal and infant PolyProfs (**B**). PolyProf alpha diversity also increases with age (**C**).

We first examined PolyProf global diversity as a function of infant age and delivery mode (Fig. 2). Like previous descriptions of taxonomy (9, 11, 44), polymorphic toxin effector/immunity profiles of infants evolve through the first year of life with a tendency toward the maternal PolyProf. PolyProf beta diversity is strongly influenced by the delivery mode, with a closer similarity to maternal PolyProf in vaginally delivered infants (Fig. 2A). Analysis of paired maternal-infant metagenomes (11, 45) showed a similar trend of gradual PolyProf convergence toward the maternal microbiome over the first year of life, quantified as decreasing Bray-Curtis dissimilarity of infant and maternal microbiomes as a function of age (Fig. 2B; Skillings-Mack test *P* < 0.001). Acquisition of new and increasing diversity of polymorphic toxin effector/immunity markers is reflected as an early-life increase in PolyProf alpha diversity (Fig. 2C; Skillings-Mack test *P* < 0.001). Using an independent data set from families in Guinea-Bissau (46), we compared PolyProf beta diversity distances by family relations (Fig. S8). Parent-child PolyProf were more similar within families than in unrelated adult-child pairs. Child-child and adult-adult distances did not differ by family status. The same pattern was observed in a large USA-based cohort of oral (saliva) microbiomes (Fig. S8) (46). The findings are consistent with vertical transmission, primarily of maternal origin, resulting in fecal and oral PolyProf similarities between related parents and children.

**Fig 3:**
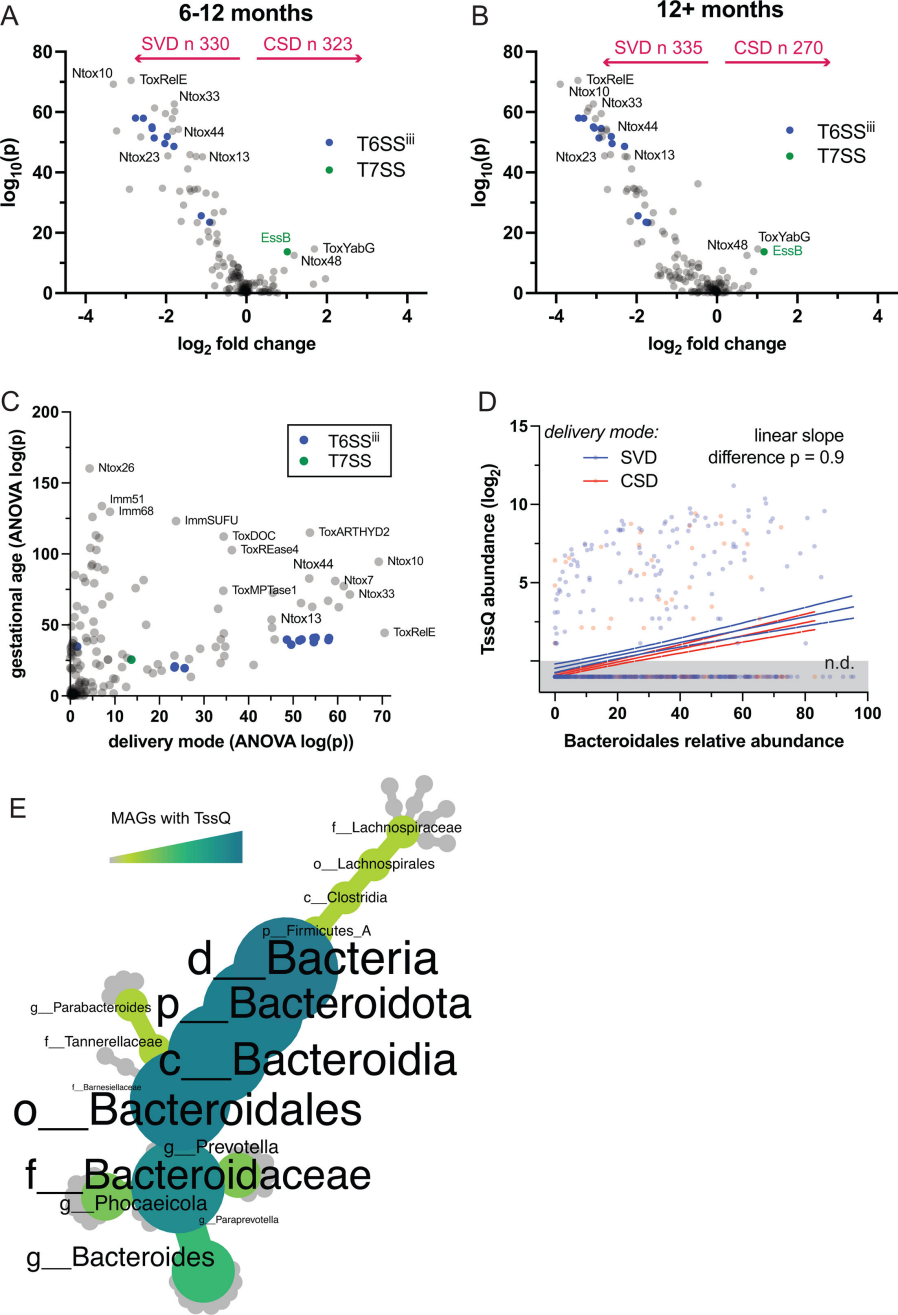
Infant delivery mode influences toxin secretion systems and effector/immunity profiles. (**A**) Vaginal delivery (SVD) is associated with enriched T6SS^iii^ and numerous effector/immunity markers, while Cesarean section delivery (CSD) metagenomes have enriched EssB (T7SS) and select toxins. The effect of delivery mode persists past 1 year of life (**B**). (**C**) Secretion system and effector/immunity markers are differentially affected by the birth delivery mode (*x*-axis) and gestational age (*y*-axis). (**D**) As a representative T6SS^iii^ marker, TssQ abundance correlates with Bacteroidales abundance. The indistinguishable linear regressions indicate a similar fraction of T6SS-positive Bacteroidales in SVD and CSD infants. (**E**) The distribution of genes with a similarity to TssQ is heavily enriched among Bacteroidales MAGs in the MGnify database, illustrated as a taxonomy plot.

In infant biospecimens collected between 6 and 12 months after delivery, there was strong enrichment of T6SS^iii^ and several effector/immunity markers in SVD, and this pattern persisted beyond 1 year of life (Fig. 3A and B; Fig. S9). PolyProf markers showed disparate dependence on the delivery mode and gestational age, analyzed with analysis of variance (ANOVA) (Fig. 3C) and MaAsLin (Fig. S9) (47). T6SS^iii^ is highly related to vaginal delivery, while the predicted RNase Ntox26 abundance is most strongly related to the subject’s age. The findings indicate the interplay of several factors in determining the toxin secretion system and effector/immunity profiles of developing microbiomes.

Depleted Bacteroidales were a prominent feature of CSD microbiomes in prior studies (10, 11). Since T6SS^iii^ is highly restricted to the order Bacteroidales, we asked whether the delivery mode dependence of this secretion system is simply reflecting the differential abundances of Bacteroidales (Fig. 3D and E). As expected, the abundance of T6SS^iii^ markers (TssQ shown as a representative) is related to Bacteroidales abundance determined using MetaPhlAn4 (37). The relationship is not linear because a minority of Bacteroidales strains carry T6SS^iii^, and quantitation by HUMAnN (“fragments per kilobase”) and MetaPhlAn (% relative abundance) is calculated differently. Linear regression was used to estimate the fraction of T6SS^iii^-positive Bacteroidales, which was not distinguishable by the delivery mode. The findings indicate that CSD infants have depleted gut microbiome T6SS^iii^ systems by virtue of lower abundances of Bacteroidales.

### Maternal effector/immunity repertoires influence newborn microbiome development

There is strong evidence for maternal-infant transmission of bacterial strains both at delivery and throughout the first year of life (12, 13, 15). Figure 2 shows PolyProf convergence with the maternal microbiome over time. We hypothesized that transmission of polymorphic effector/immunity and secretion system-encoding strains from the mother to infant influences microbiome development, and we predicted that the presence of these markers in the maternal microbiome would strongly predict detection in the infant. To address this, we focused on infants with paired maternal metagenomes (11, 45).

**Fig 4:**
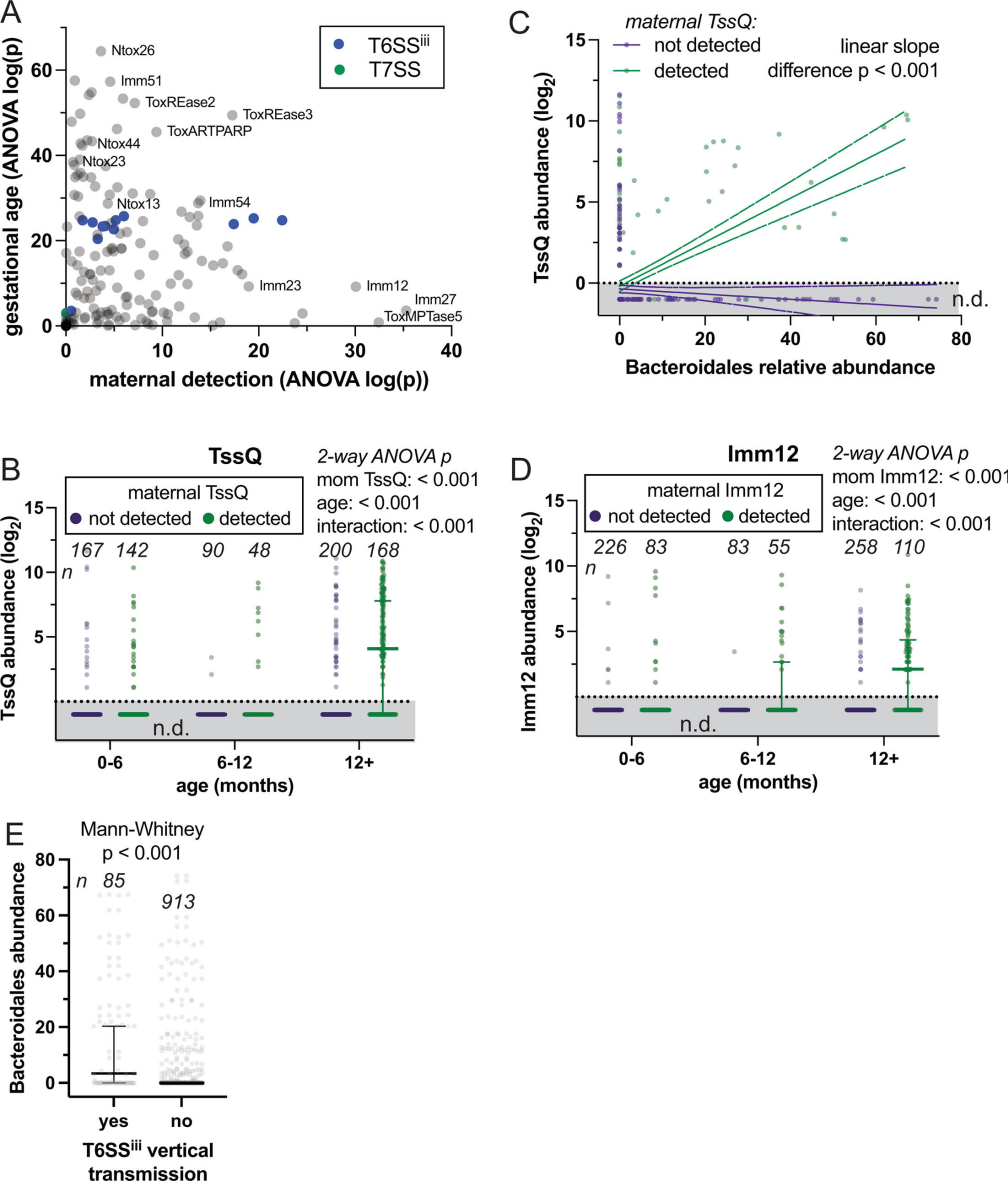
Maternal secretion systems and effector/immunity genes predict profiles of infants. (**A**) The abundance of T6SS^iii^ and many effector/immunity markers in infant metagenomes is related to the same marker being carried by the mother (*x*-axis) and to gestational age (*y*-axis). (**B**) The T6SS^iii^ representative marker, TssQ, is more abundant in infants born to mothers with TssQ, and this difference increases with age. (**C**) Linear slope represents a correlation between TssQ abundance and Bacteroidales abundance in metagenomes. A higher fraction of Bacteroidales are TssQ-positive in metagenomes of infants born to mothers with TssQ. (**D**) Imm12 as a representative immunity marker is also related to both maternal Imm12 detection and infant age. (**E**) Bacteroidales are more abundant in infants with vertical transmission of TssQ (mother and infant both positive) than those without.

T6SS^iii^ systems and several effector/immunity markers in infant metagenomes were strongly related to their detection in the maternal metagenomes (Fig. 4A; Fig. S10 [MaAsLin]). TssQ as a representative of T6SS^iii^ was more abundant in metagenomes of infants born to mothers whose microbiomes also carry TssQ, and this difference increased through the first year of life (two-way ANOVA *P* < 0.001; Fig. 4B). A higher fraction of Bacteroidales carried TssQ in infants born to mothers with detectable TssQ than those without, illustrated as a linear slope difference (*P* < 0.001; Fig. 4C). Bacteroidales were more abundant in the metagenomes of infants with likely vertical transmission (TssQ-positive mother and infant) than those without (Fig. 4E). This finding suggests that T6SS^iii^ confers an advantage to Bacteroidales over other taxonomic groups. However, the data are correlative and cannot establish a causal relationship by itself. Other effector/immunity markers, such as Imm12, show a similar pattern of higher abundance in infants whose mother’s microbiome carries the same gene (Fig. 4D). Imm12 immunity is frequently encoded adjacent to and predicted to protect against Tox-URI2 effectors and predicted DNases (7). Imm12 and other effector/immunity markers correlating to maternal detection are not restricted to Bacteroidales. The findings provide strong evidence for maternal-infant transmission of the polymorphic secretion system and effector-/immunity-encoding bacteria. The time course patterns suggest that transmission continues through the first year of life.

### Breastfeeding changes effector/immunity profiles

Secretion system and effector-/immunity-encoding strain transmission from the mother to infant continues to occur after SVD, and prior research has shown that breastfeeding is an important mode of microbiome vertical transmission (15). We asked whether breastfeeding status also affects PolyProf profiles in the infant microbiome. To address this, we focused analysis on SVD infant metagenomes stratified by breastfeeding status (11, 45, 48).

**Fig 5:**
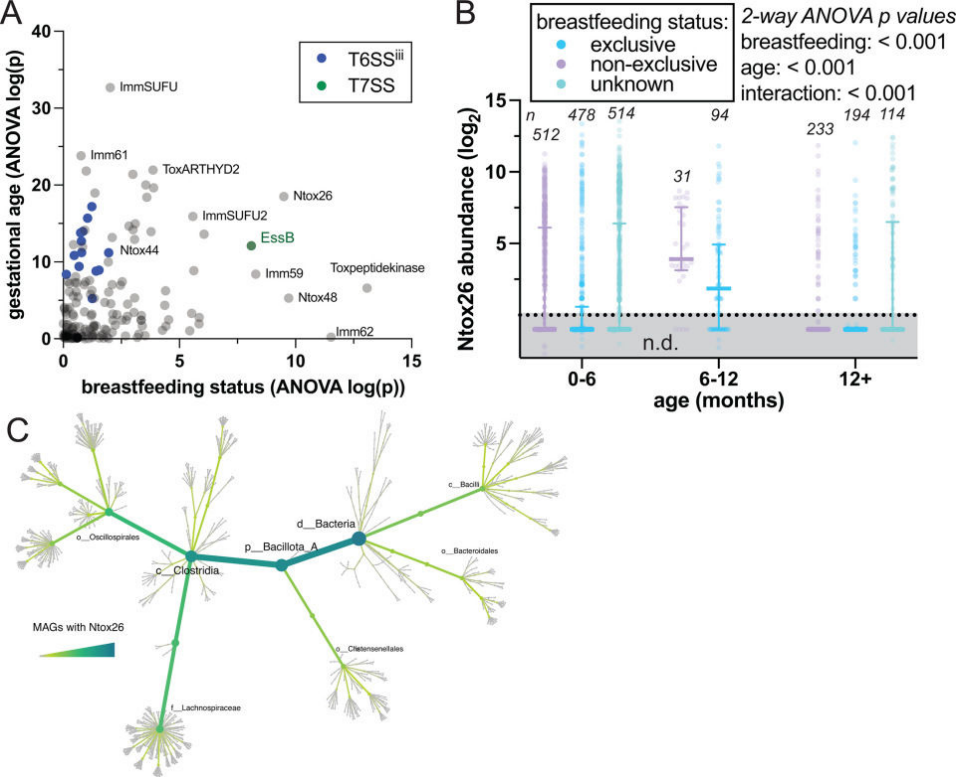
Breastfeeding affects infant microbiome effector/immunity profiles. (**A**) Some effector and immunity markers were significantly related to breastfeeding status and gestational age. (**B**) Ntox26 abundance is lower in exclusively breastfed infants throughout the first year of life than in non-exclusive breastfeeding or infant metagenomes with unknown nutritional sources. (**C**) Ntox26 is found predominantly in Bacillota MAGs.

The T7SS structural protein EssB and several effector/immunity markers were significantly associated with breastfeeding status and gestational age (Fig. 5A; Fig. S11 [MaAsLin]). Ntox26 is shown as an example effector that was lower in abundance in exclusively breastfed infants than in non-exclusively breastfed or unknown status infant microbiomes (Fig. 5B). Both Ntox26 and EssB (T7SS) are predominantly distributed among Bacillota MAGs. We conclude that breastfeeding also correlates with infant PolyProf profiles during the first year of life, although the relationships are smaller in magnitude than the delivery mode and maternal profiles.

### Effector/immunity profiles are related to social strain sharing

Close contact is likely an essential requirement for vertical strain transmission. Recent evidence suggests that gut bacterial strains are also shared during social interactions (40). We hypothesized that polymorphic toxin effector-/immunity-encoding strains may have a competitive advantage in establishing colonization during social contact strain sharing. If the hypothesis is correct, we expect that PolyProf would be more similar among socially connected individuals and correlate with strain sharing.

**Fig 6:**
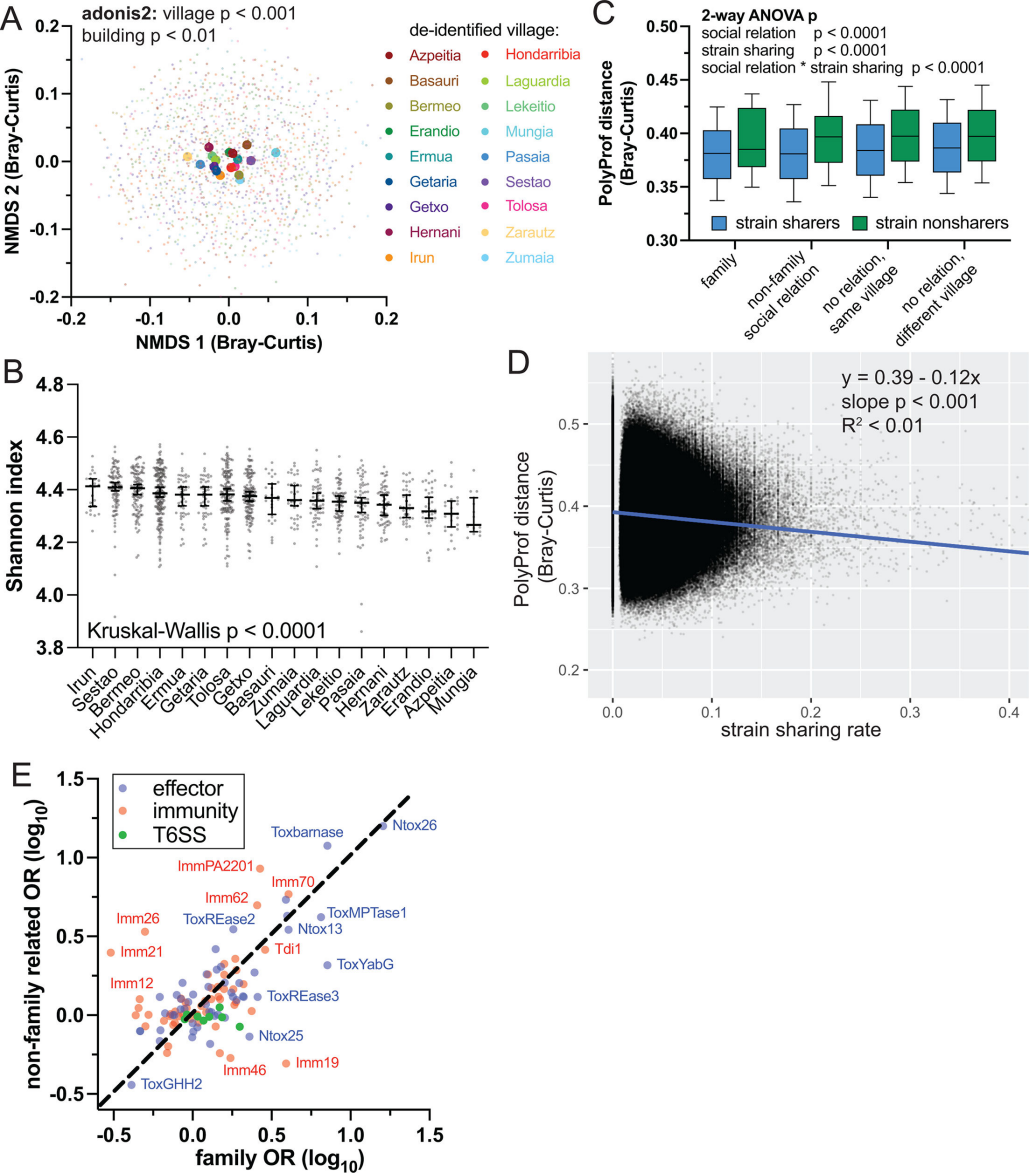
PolyProf diversity correlates with social contacts and strain sharing. (**A and B**) PolyProf beta and alpha diversity in a Honduras cohort are distinct by village and building identifiers. (**C**) PolyProf Bray-Curtis distance, calculated for every sample pair, is strongly related to strain sharing and the specific social relationship. Family relatives and non-family social contacts have the most similar PolyProf, particularly when strain sharing is detected. (**D**) PolyProf Bray-Curtis distance shortens as strain sharing increases. (**E**) Some effector/immunity genes tend to be detected in both subjects when strain sharing is detected, quantified as log odds ratios. Markers falling on the *y* = *x* line, such as Ntox26, are equally co-detected in family and non-family social contacts. Scatter to the lower right indicates a stronger association with family strain sharing, as for the T6SS^iii^ markers.

We applied PolyProf to 1,117 fecal metagenomes from subjects living in isolated villages in Honduras (40). PolyProf alpha and beta diversity clustered by co-residency in a village or building and high strain sharing rates between individuals correlated with more similar PolyProf (Fig. 6). PolyProf beta diversity was most similar for subject pairs within a family, followed by non-family social contacts and village co-residents (Fig. 6C). Subject pairs in all social relation groups had more similar PolyProf when strain sharing was detected (Fig. 6C and D; two-way ANOVA *P* < 0.001). We conclude that PolyProf similarity is highly correlated with strain sharing among social contacts. Although the strongest relationships are seen for family and close social contacts, PolyProf patterns are distinguishable on the village level. Thus, social context is a key determinant of PolyProf on the population (village-wide) level.

We next asked which specific effector/immunity genes are most highly correlated with social strain sharing. We reasoned that marker genes promoting strain transmission will be more likely co-detected in subject pairs that share strains (donor and recipient). From 2 × 2 tables of effector/immunity co-detection and strain sharing detection, odds ratios (OR) were calculated and tested with Fisher’s exact statistics (Fig. 6E). Most effector/immunity markers were either neutral (OR ~ 1) or more frequently co-detected in strain sharers (OR > 1). Some markers were co-detected in strain sharers with specific types of social interaction. For example, Imm 21 co-detection is enriched in non-family social contacts but not in families. Consistent with the prior mother-infant data (Fig. 4), T6SS^iii^ promotes strain sharing within families (reflecting vertical transmission) but does not correlate with non-family strain sharing (co-detection ORs ~ 1; Fig. 6E). Since all subjects in the cohort are adults, the results indicate the persistence of T6SS^iii^ effects on transmission beyond infancy and childhood. The most highly strain sharing-associated marker, Ntox26, was also prominently associated with vertical transmission during microbiome development (Fig. 3 to 5), collectively suggesting this effector is related to strain transmission in multiple contexts.

To identify strain-level PolyProf and secretion system features related to strain sharing, we assembled 130,852 MAGs from Honduras cohort metagenomes and calculated their relative transmissibility between individuals. By applying TXSScan (49) to all MAGs and associating the detection of secretion system genes with transmissibility, we identified two systems positively associated with transmission: Tad and flagellum (Fig. S12 through 14). These two systems have diverse known functions that include, but are not limited to, effector secretion. For example, a Tad-like system in *Myxococcus xanthus* mediates contact-dependent interbacterial antagonism (50, 51). The flagellar type III secretion system exports proteins for flagellum assembly and is also known to secrete antimicrobial toxins in some strains (52). Three predicted RNAse effector classes, Ntox13, Ntox23, and Ntox44, were enriched in highly transmissible MAGs, detected with gene set enrichment analysis (Fig. S14). All three effector types were also significantly associated with infant microbiome development and vertical transmission (Fig. 3 to 5), implicating these effectors as related to strain transmission in multiple contexts.

## DISCUSSION

We have developed and applied a marker sequence-based approach to profile polymorphic toxin secretion systems and effector/immunity classes in metagenomes. These systems are known to mediate interbacterial antagonism within the intestinal ecosystem (25, 31), but little was previously known about their relationship to dysbiosis and human disease. A conceptual advance of this study is combining existing marker sequence profiling tools and a new microbiome-focused effector/immunity sequence database to create and validate a tool for profiling metagenomes. A central finding of this study is that PolyProf biomarkers readily distinguish adult human disease states, which suggests that polymorphic toxins are perturbed in disease dysbiosis-specific ways. We speculate that many of the observed associations are effects of the disease state rather than causes. For example, intestinal inflammation in IBD drastically changes the bacterial microenvironment with the release of oxygen species, mucosal disruption and bleeding, and compromise of mucin barriers (5). In the dysbiotic state, interbacterial interactions are also perturbed (53, 54), which may result in competitive advantages (or disadvantages) of certain effectors and corresponding immunity proteins. Our study highlights several disease-associated markers that suggest avenues for mechanistic studies in disease models to address the directionality of causation. The performance of machine learning models based solely on PolyProf is comparable to that of similar approaches using bacterial taxonomy to predict disease states (41). Combining PolyProf and bacterial taxonomy enabled the development of more accurately predictive models. This finding emphasizes the importance of strain-level functional characteristics like secretion systems in disease dysbiosis, which are not fully accounted for with traditional metagenomic taxonomy analyses. There is substantial promise for enhanced disease-related classifiers that incorporate the quantification of functionally important genes in combination with taxonomy.

Our second major finding is that polymorphic toxin secretion systems and effector/immunity profiles are highly dynamic and variable in the developing microbiome of infants. Exposure to maternal bacteria at delivery (vaginal vs surgical), vertical strain transfer, and breastfeeding status all shape the PolyProf profile diversity through the first year of life. In one example, we show that the presence of T6SS^iii^ is related to the abundance of Bacteroidales and could play a role in shaping microbiome taxonomic diversity. As Verster et al. have modeled and demonstrated experimentally in *Bacteroides fragilis*, T6SS^iii^ effectors mediate antagonism in the intestine and exclude related, non-immune Bacteroidales from colonization (32, 55). Thus, vertical transmission of bacteria with secretion systems and immunity to the dominant effectors is likely one mechanism by which developing newborn microbiomes gravitate toward the parental microbiome, excluding competing bacteria to which the infant is exposed. Our study focused on the dominant role of maternal-infant transmission because of data availability, but recent studies indicate stable transmission from paternal sources as well (14). We raise the hypothesis that vertical polymorphic effector immunity transmission plays a role in the non-genetic inheritance of disease. Seminal studies in the microbiome of obesity, for example, have demonstrated that the transfer of the microbiome from obese individuals to mice is sufficient to promote weight gain (2). Does the dysbiotic microbiome and PolyProf of a parent with obesity get transmitted to the offspring and increase the risk for obesity later in life?

Roles for polymorphic toxin secretion systems in microbiome development are not restricted to the first year of life. Our third major finding is that PolyProf is related to strain sharing during social interactions. Beghini et al. showed that gut microbiome strain sharing among a cohort of people living in isolated villages in Honduras occurs through both familial and non-familial social interactions (40). We find that PolyProf beta diversity and specific effector/immunity genes are, in turn, related to strain sharing, not only in families but also with close social contacts and village co-residents. These combined studies indicate the ongoing exchange of commensal bacteria through human social interactions after the first year of life. Some polymorphic toxin secretion system effector and immunity genes may even influence strain sharing, likely through competitive colonization mechanisms.

### Conclusions

PolyProf accurately quantifies polymorphic toxin secretion effector/immunity genes in shotgun metagenomics data sets, distinguishes many disease states, and can be used to construct high-performing machine learning models to predict disease. Polymorphic toxin secretion effector/immunity genes correlate with parent-child vertical strain transmissions and strain sharing among non-family social contacts. In summary, this study adds correlational evidence that polymorphic toxin secretion systems, effectors, and immunity proteins are important determinants of initial microbiome development and social strain sharing, and they are markers of diverse adult disease statuses. Further study is needed to determine their mechanistic roles in disease and for the development of potential microbiome-based diagnostics.

### Limitations

Metagenomic profiling is correlative and cannot by itself establish causal relationships between TSS and diseases, microbiome development, or strain sharing. The results and conclusions of the meta-analysis are somewhat dependent on the accuracy of the metadata. For example, the “healthy controls” of an IBD study may include tobacco users and confound the smokers vs all healthy controls analysis. However, the expected effect would be increased variance in the control group and bias toward the null hypothesis (smokers and healthy controls not distinguishable) and therefore lead to underestimation of disease-specific effects and lowered performance of disease classifiers. As in all meta-analyses, population and methodological differences could affect the cross-study comparisons. While marker sequences are a well-established method for gene family quantification, off-target mapping could increase the variance and bias toward the null hypothesis for any marker family. The marker sequence database derived from ~200,000 MAGs is a substantial advance toward representing the diversity of effector/immunity sequences in gut microbiomes, but it is biased toward the populations, metagenomes, and derived MAGs of the original studies. As a result, PolyProf may not detect all effector/immunity class members. The power and accuracy of machine learning models are dependent on sample size, uniformity, and specific parameters employed. The parameters used in this study were optimized for IBD and then applied to all other comparisons for uniformity and comparability. Better-performing models are certainly achievable.

## MATERIALS AND METHODS

### Data and software resources, machine learning classifier generation, and testing

See Supplemental methods for details.

### Disease-related dysbiosis comparisons

PolyProf profiling was performed on 14,740 human gut metagenomes using HUMAnN 3 (37) and default settings other than input of the PolyProf custom protein database and bypass of the nucleotide search. Analyses were performed on relative abundance values assigned by HUMAnN, which are described as “reads per kilobase” (37). Non-detection was assigned a value of 0.5 for downstream statistical testing. Polymorphic effector/immunity beta diversity was compared within each study set, as defined in Table S2. Exceptions were osteoarthritis, obesity, and rheumatoid arthritis studies that did not include non-disease control groups. Individual disease-distinguishing markers were identified using multivariate logistic regression of marker relative abundances with diagnosis as the dependent variable. Beta diversity was measured as Bray-Curtis dissimilarity and alpha diversity as Shannon index using the vegan R package. The meta-analysis design before beginning analysis was to perform 3-tiered comparisons: (i) compare diagnosis groups within each study, (ii) compare diagnosis groups to all “healthy controls,” and (iii) compare diagnosis groups to all other metagenomes. Each comparison included Shannon index alpha diversity testing with Mann-Whitney or Kruskal-Wallis tests, as appropriate, Bray-Curtis beta diversity distance testing with adonis2 (vegan R package), and LefSe linear discriminant analysis (56). Global beta diversity was visualized using NMDS analysis of Bray-Curtis distance matrices. All pairwise comparison statistical tests were adjusted for FDR 0.01 using the Benjamini-Hochberg method. Within the IBD cohort, 662 patients had available fecal calprotectin data. Calprotectin was linearly correlated with each PolyProf marker using the corr package in R.

### Infant delivery mode, maternal-infant transmission, and breastfeeding status microbiome analysis

PolyProf profiling and taxonomic quantitation with MetaPhlAn4 (57) were applied to all infant and maternal samples. For delivery mode analyses, only infant microbiomes with metadata indicating the delivery mode were included. Differences in marker gene abundances were calculated as log_2_ fold change using median values and pairwise statistical testing using Student’s *t*-tests, visualized as a volcano plot. Effects of delivery mode and gestational age were measured using ANOVA. Infant ages were binned as 0–6 months, 6–12 months, or >12 months. Marker sequence changes were quantified over these age bins and stratified by the delivery mode. Statistical testing was a two-way ANOVA. Mixed-effects modeling with MaAsLin 3 was also performed, adjusting for gestational age as a continuous variable (47). For maternal-infant transmission studies, only infant metagenomes with a linked maternal metagenome (11) were included. Any non-zero marker gene quantity in the maternal metagenome was defined as detection. Time course paired analysis of PolyProf diversity was tested using a generalization of the Friedman test for missing data called the Skillings-Mack test (58).

### Social strain sharing microbiome analysis, metagenome-assembled genomes, MAG transmissibility, TXSS, and PolyProf analysis

See Supplemental methods for details.

## Data Availability

Source metagenomic data are publicly available from the Sequence Read Archive, and accession numbers are given in Table S2. The PolyProf database, sequences, and associated graphical data are available on GitHub at https://github.com/dustin-bosch/PolyProf/.

## SUPPLEMENTAL MATERIAL

Supplemental figures msystems.00305-26-s0001.pdf Figures S1-S14.

Supplemental tables msystems.00305-26-s0002.xlsx Tables S1 and S2.

Supplemental methods msystems.00305-26-s0003.pdf Additional experimental details.

ASM does not own the copyrights to Supplemental Material that may be linked to, or accessed through, an article. The authors have granted ASM a non-exclusive, world-wide license to publish the Supplemental Material files. Please contact the corresponding author directly for reuse.
