# Supplementary material for: Metagenomic polymorphic toxin effector and immunity profiling predicts microbiome development and disease-related dysbiosis
Source: mSystems. 2026 May 22;11(6):e00305-26. doi: 10.1128/msystems.00305-26 (PMC13288982; doi:10.1128/msystems.00305-26)
Supplement: Supplemental figures — Figures S1-S14. [file msystems.00305-26-s0001.pdf]

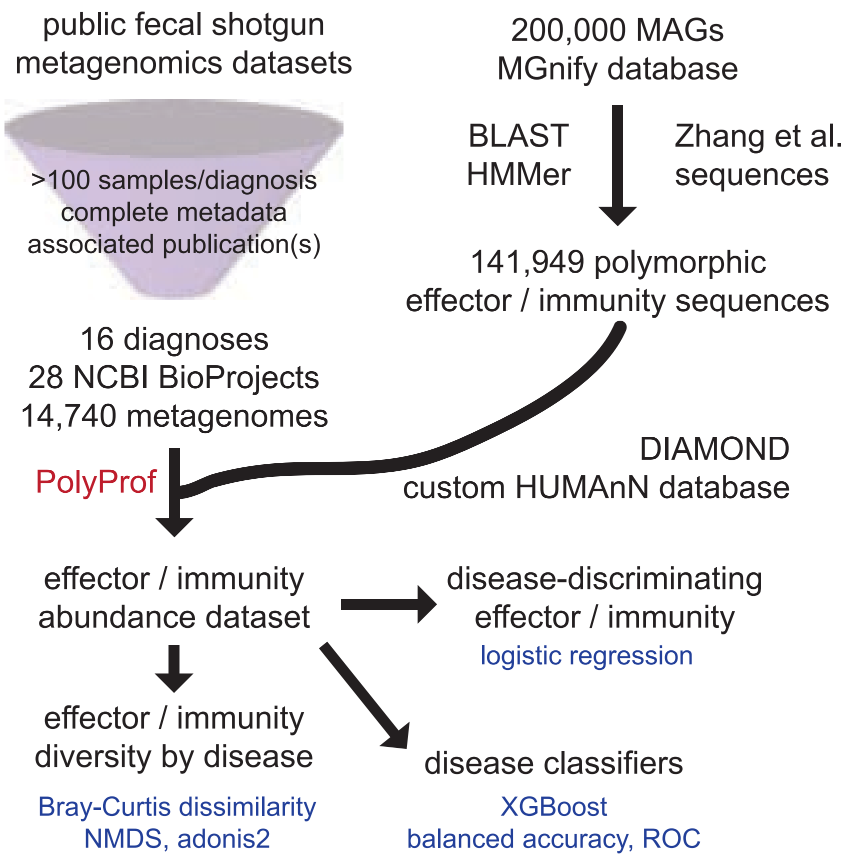

Figure S1. *Polymorphic toxin effector / immunity profiling meta-analysis design.* Fecal metagenomic sequence data was profiled using a custom HUMAnN database of MAG-derived effector / immunity protein sequences. Effector / immunity abundance profiles were analyzed to identify disease-specific associations.

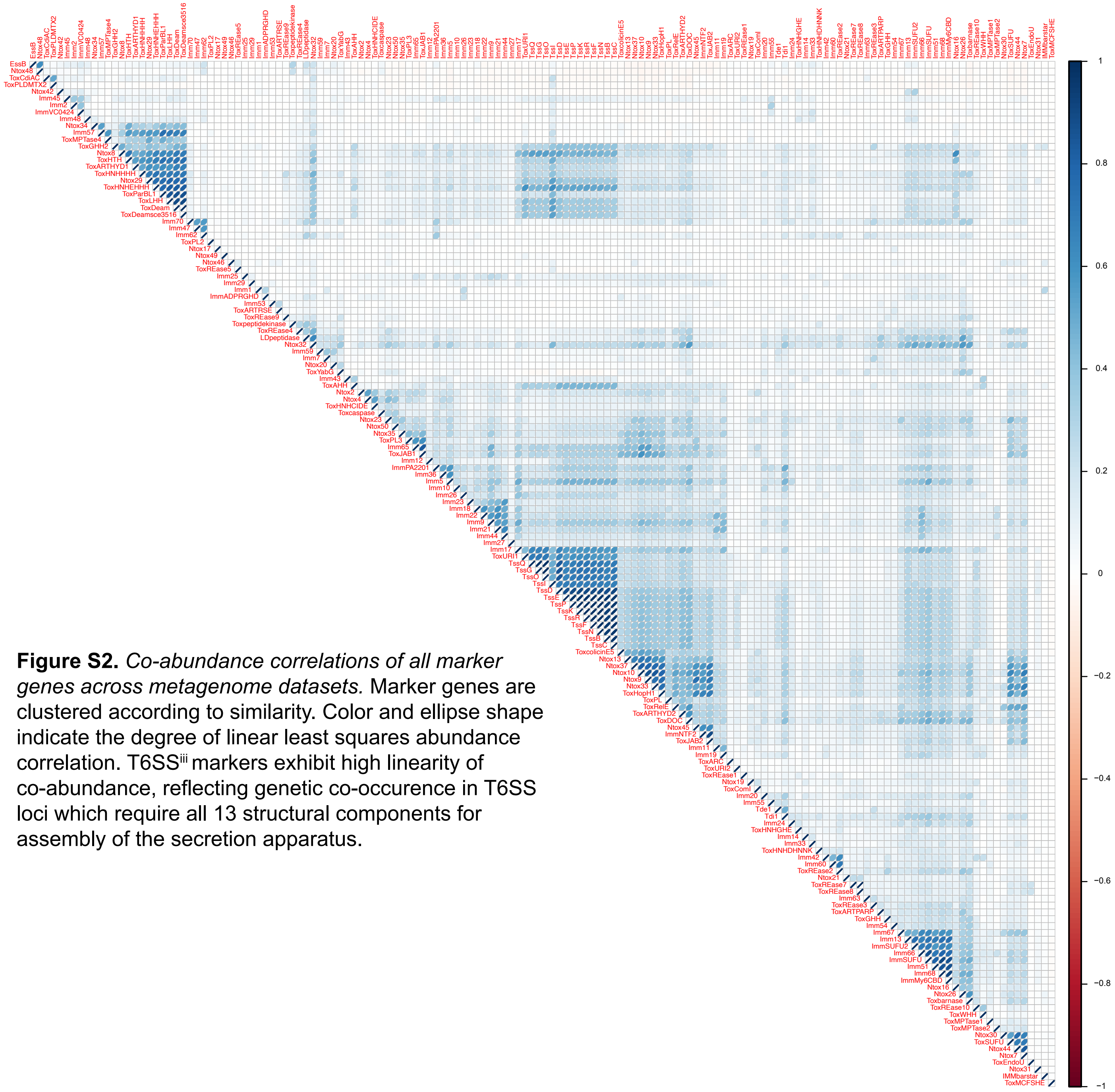

Figure S2. *Co-abundance correlations of all marker genes across metagenome datasets.* Marker genes are clustered according to similarity. Color and ellipse shape indicate the degree of linear least squares abundance correlation. T6SS[iii] markers exhibit high linearity of co-abundance, reflecting genetic co-occurence in T6SS loci which require all 13 structural components for assembly of the secretion apparatus.

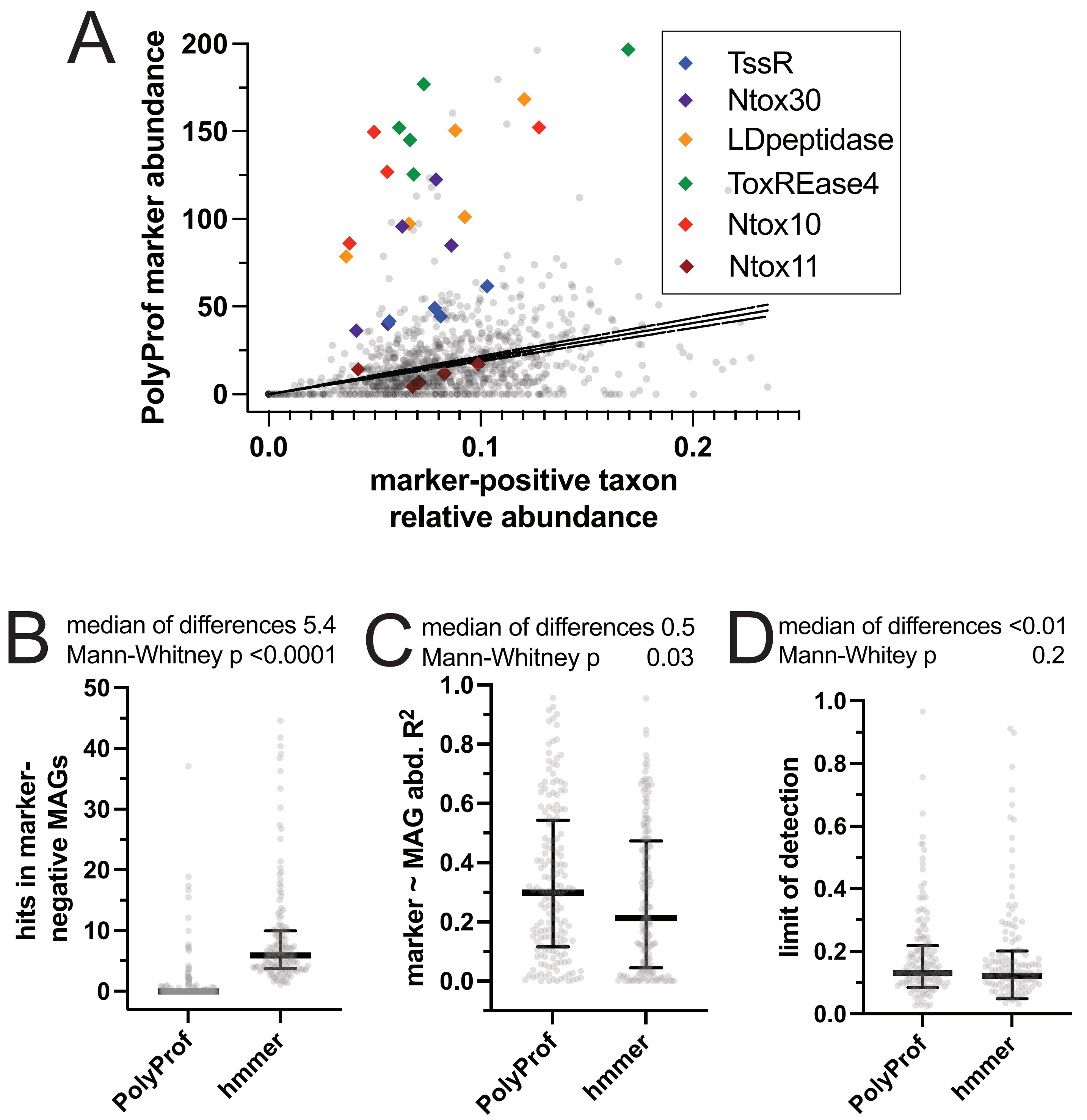

Figure S3. *PolyProf marker performance on simulated metagenomic data.* Metagenomic data were simulated from MAGs with and without known corresponding effector / immunity genes. (A) Strong linear correlation between simulated taxon abundance and PolyProf marker abundance was observed (black linear regression line with dashed 95% confidence intervals). Six markers had marker abundance measurements that deviated significantly from the expected value based on taxon abundance, highlighted with colored diamonds. (B) PolyProf had fewer off-target hits in marker-negative MAGs compared to hidden Markov models generated from Zhang et al's effector / immunity sequences. (C) PolyProf abundances also had slightly better linear correlation with the corresponding marker-positive MAG abundance than the HMM approach. (D) Limit of detection estimates (sensitivity) were indistiguishable between methods.

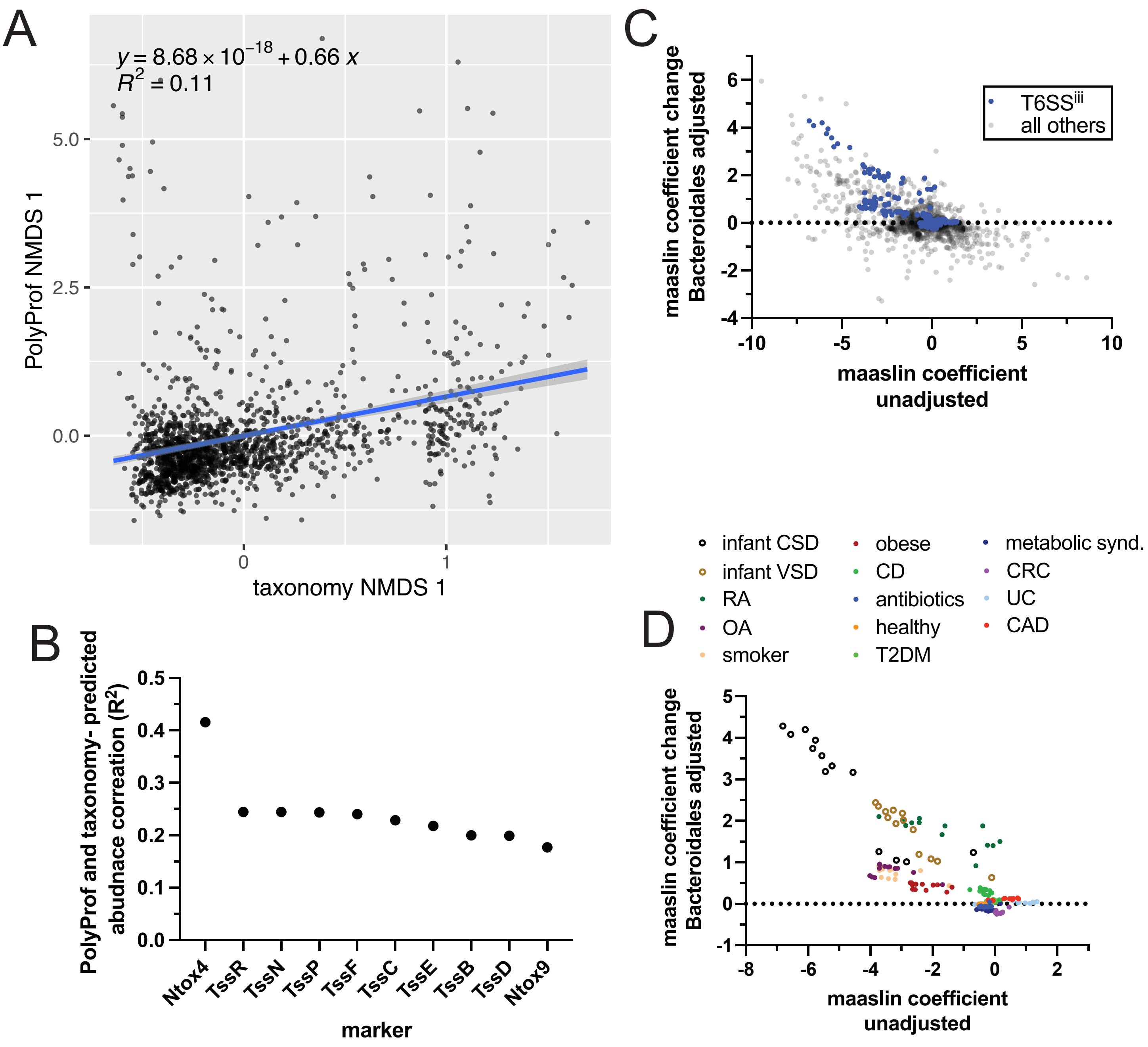

Figure S4. *PolyProf variance is weakly related to taxonomic beta diversity.* Metagenomes (n = 1537) from the IBD cohort including healthy controls were analyzed with PolyProf and MetaPhlAn, followed by Bray-Curtis dissimilary NMDS analysis. (A) The dominant PolyProf and taxonomy NMDS values for each metagenome are fitted with linear regression. The R^2 value of 0.11 indicates that approximately 11% of PolyProf variance is explained by taxonomic beta diversity. (B) Linear regression models for each marker comparing taxonomy-based predicted and actual PolyProf-measured abundances (plots available on GitHub). Variance explained by taxonomy (R^2) is shown for the 10 most highly taxonomy-correlated markers. Ntox4 abundance is exceptionally predictable from taxonomy, followed by eight components of the Bacteroidales-restricted T6SS^{iii}. (C) Associations of PolyProf marker abundances with diagnoses were calculated using MaAsLin3, with and without adjustment for Bacteroidales abundance. The differences in model coefficients upon Bacteroidales adjustment are plotted vs. the unajusted coefficients. A y-axis value of zero would indicate no effect of Bacteroidales abundance on PolyProf-disease associations. Some T6SS^{iii} diagnosis associations are influenced by taxonomy. (D) To identify specific diagnoses where T6SS^{iii} abundance is driven by taxonomy, a similar plot shows only T6SS^{iii} maker associations, colored by diagnosis. For some populations, such as infants (open circles) T6SS^{iii} is highly affected by Bacteroidales abundance. Other diagnoses, such as UC and CAD, haveT6SS^{iii} maker enrichment that is largely independent of Bacteroidales abundance.

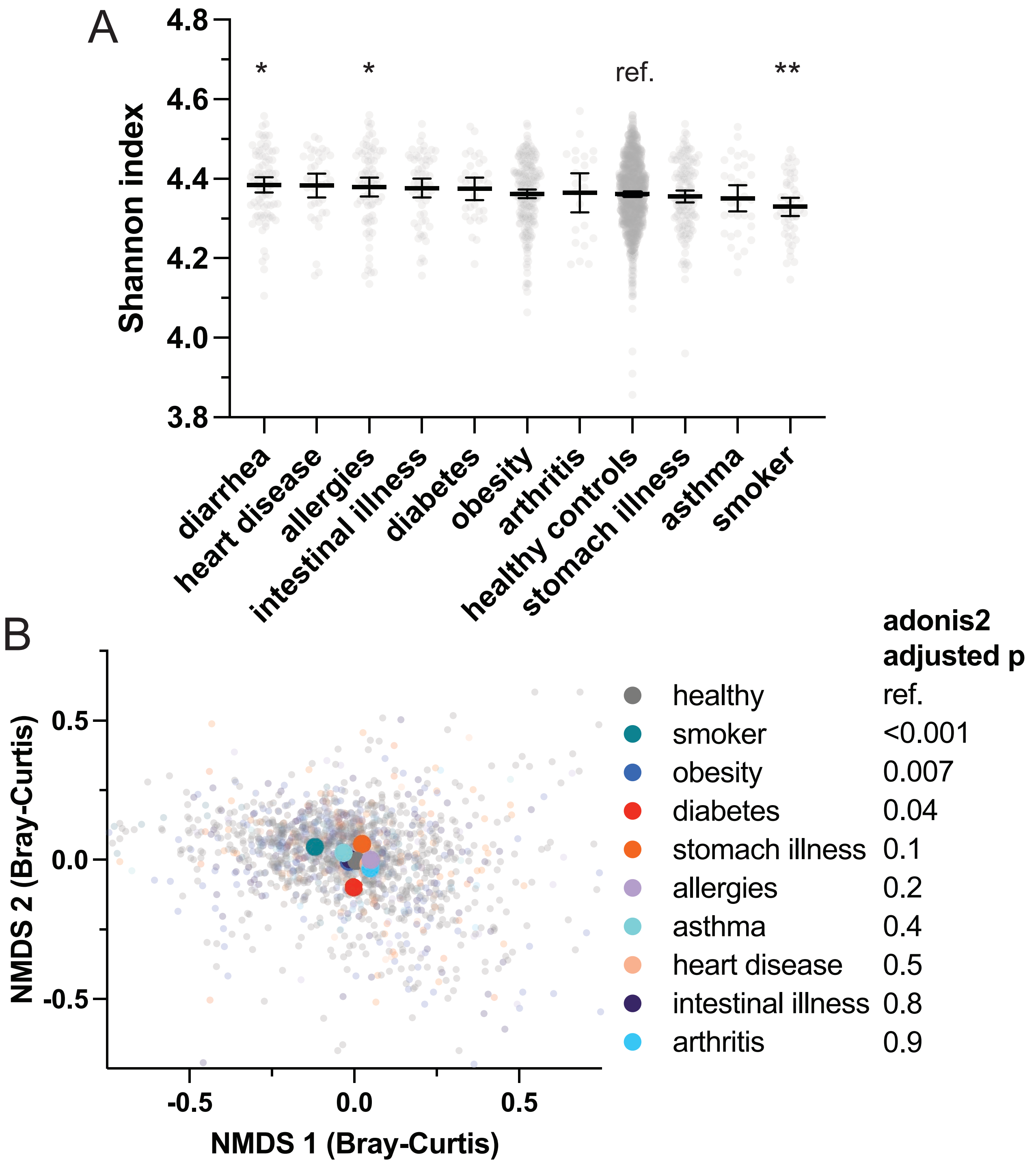

Figure S5. *PolyProf diversity correlates with disease state in an independent Honduras cohort.* A) Alpha diversity was compared by participant self-reported disease, symptoms, or tobacco use. Obesity status was assigned based on an objectively measured BMI > 30 kg/m^2. Diarrhea, allergies, and smoking were associated with significant alpha diversity deviation from healthy controls (* $p < 0.05$ or ** $p < 0.01$, adjusted for multple comparisons). B) PolyProf beta diversity also differed by disease, with strongest contributions from smoking, obesity, and diabetes in multivariate adonis2 testing.

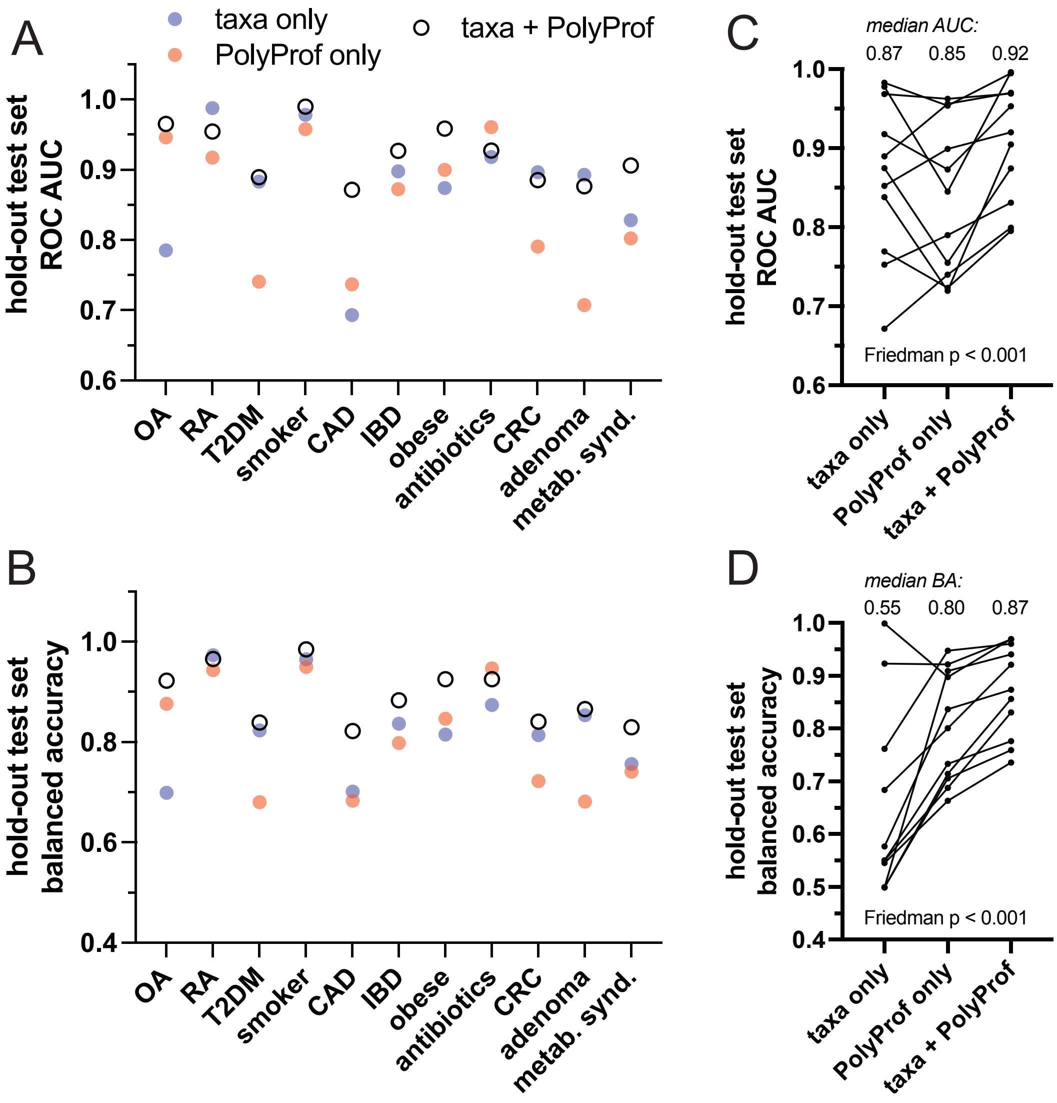

Figure S6. *Elastic net performance for healthy microbiomes and the additve contributions of PolyProf and taxonomy.* (A, B) ROC area under the curve and balanced accuracy are calculated for elastic nets trained to distinguish each diagnosis from all healthy controls. (C, D) Performance statistics for the models in Figure 1 E-F are compared between taxonomy only, PolyProf only, and combined data input. Combined data models had significantly higher AUC and balanced accuracy.

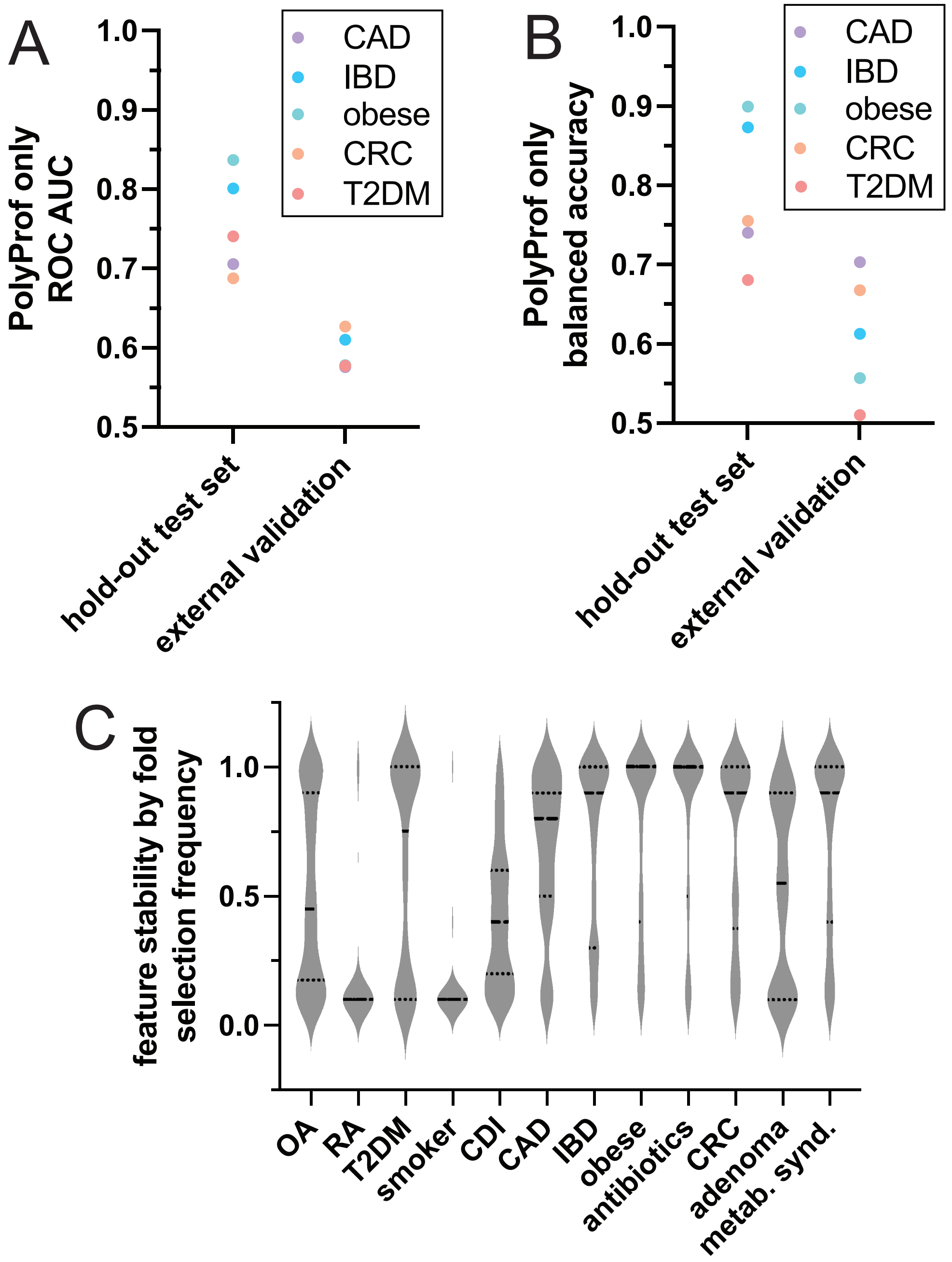

Figure S7. *External dataset validation for PolyProf elastic net models.* (A, B) Disease vs. all other diagnoses elastic net models in Figure 1 were validated using random hold-out test sets and independent datasets, obtained from curatedMetagenomicData. As expected, performance on external datasets was lower for all models, but still >0.5. (C) Feature selection frequencies over 100 folds are represented as violin plots. Lines indicate median and quartiles.

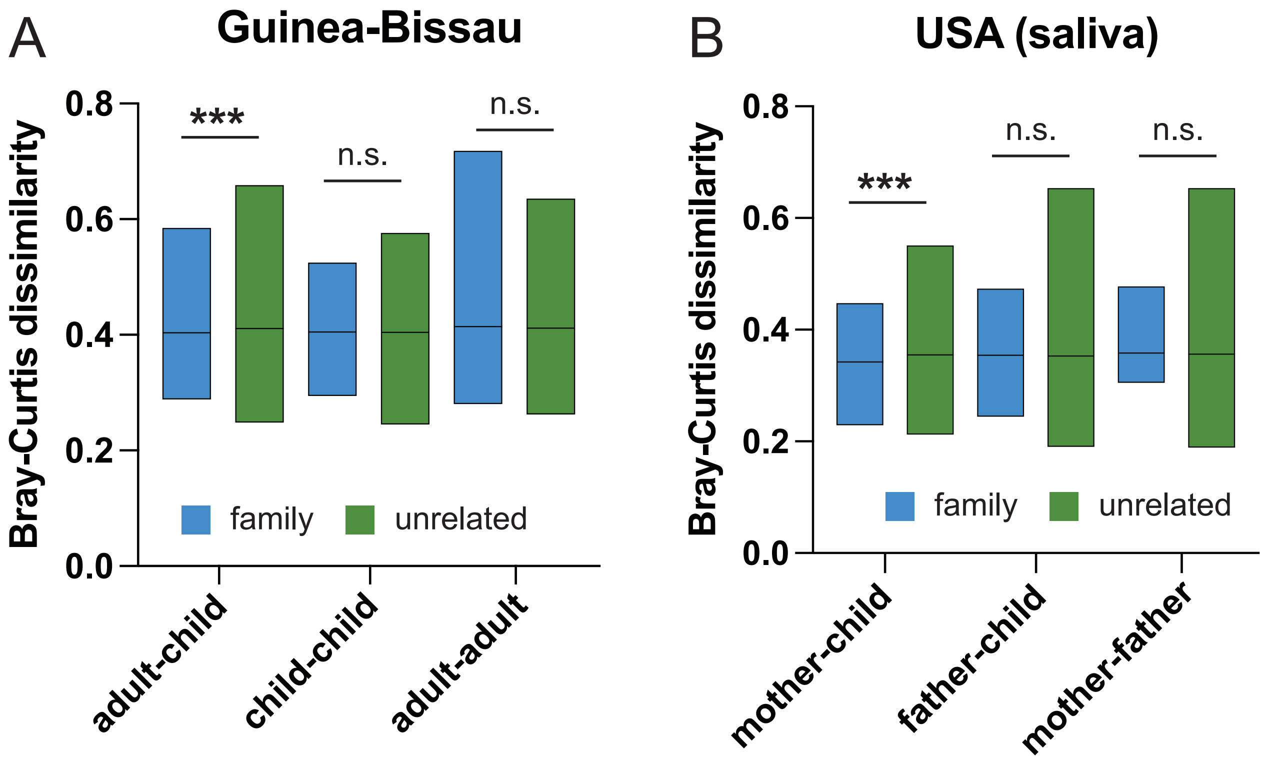

Figure S8. *Fecal and oral microbiome PolyProf are similar in related mother-child pairs.* (A) In a fecal microbiome dataset from a Guinea-Bissau population, PolyProf beta diversity differences measured by Bray-Curtis dissimilarity were lower in related parent-child pairs. Between-child and -adult distances were not different according to family relationships. (B) Oral microbiomes from a USA cohort were most similar in related mother-child pairs. Boxplots represent median and interquartile range. *** $p < 0.001$ Mann-Whitney test. n.s. not significant.

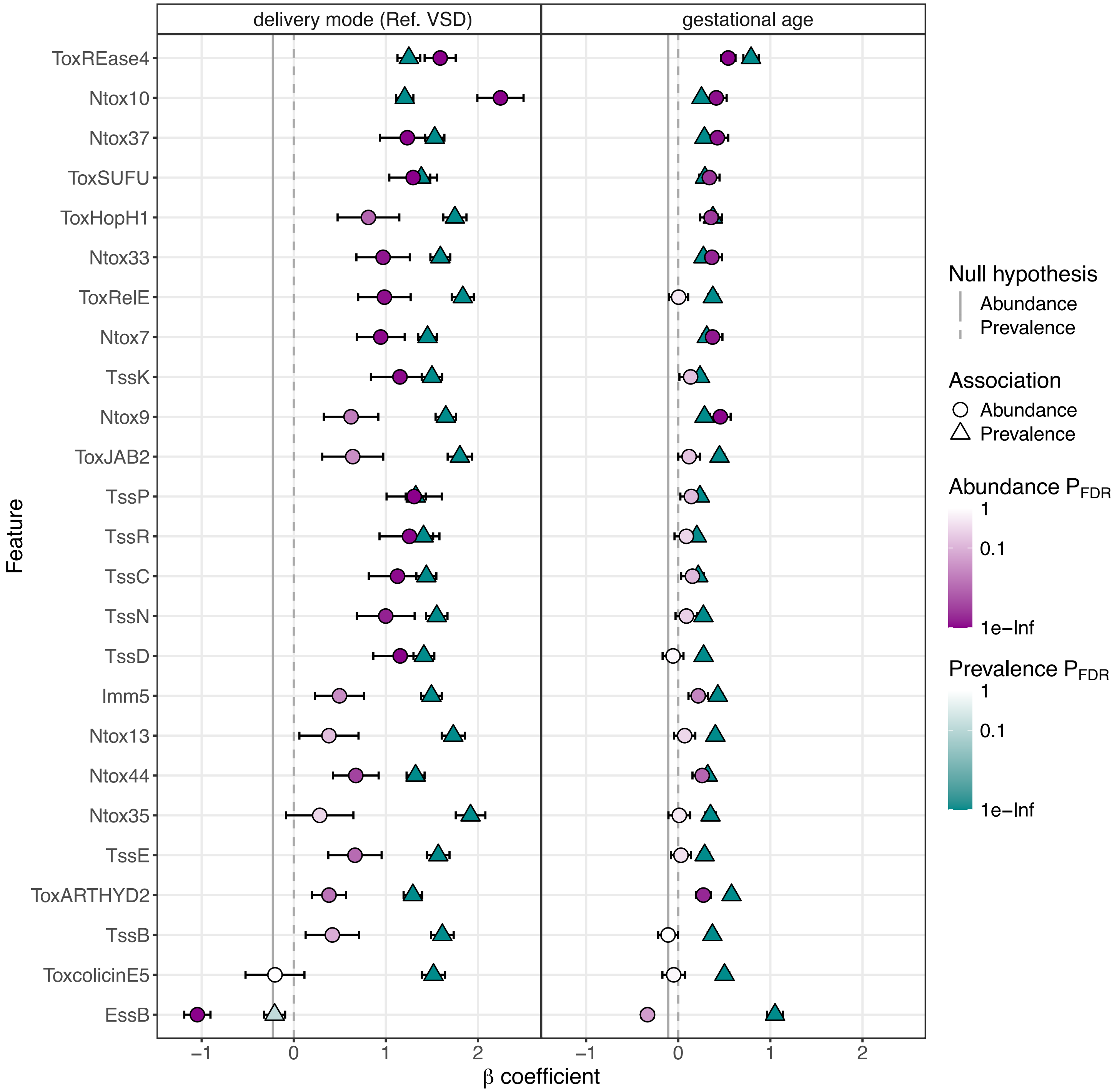

Figure S9. *MaAsLin marker gene analysis by delivery mode and gestational age.* Data corresponding to Figure 3 were assessed with mixed-effects modeling (MaAsLin 3) using numerical gestational age and delivery mode (CSD vs. SVD) as fixed effects.

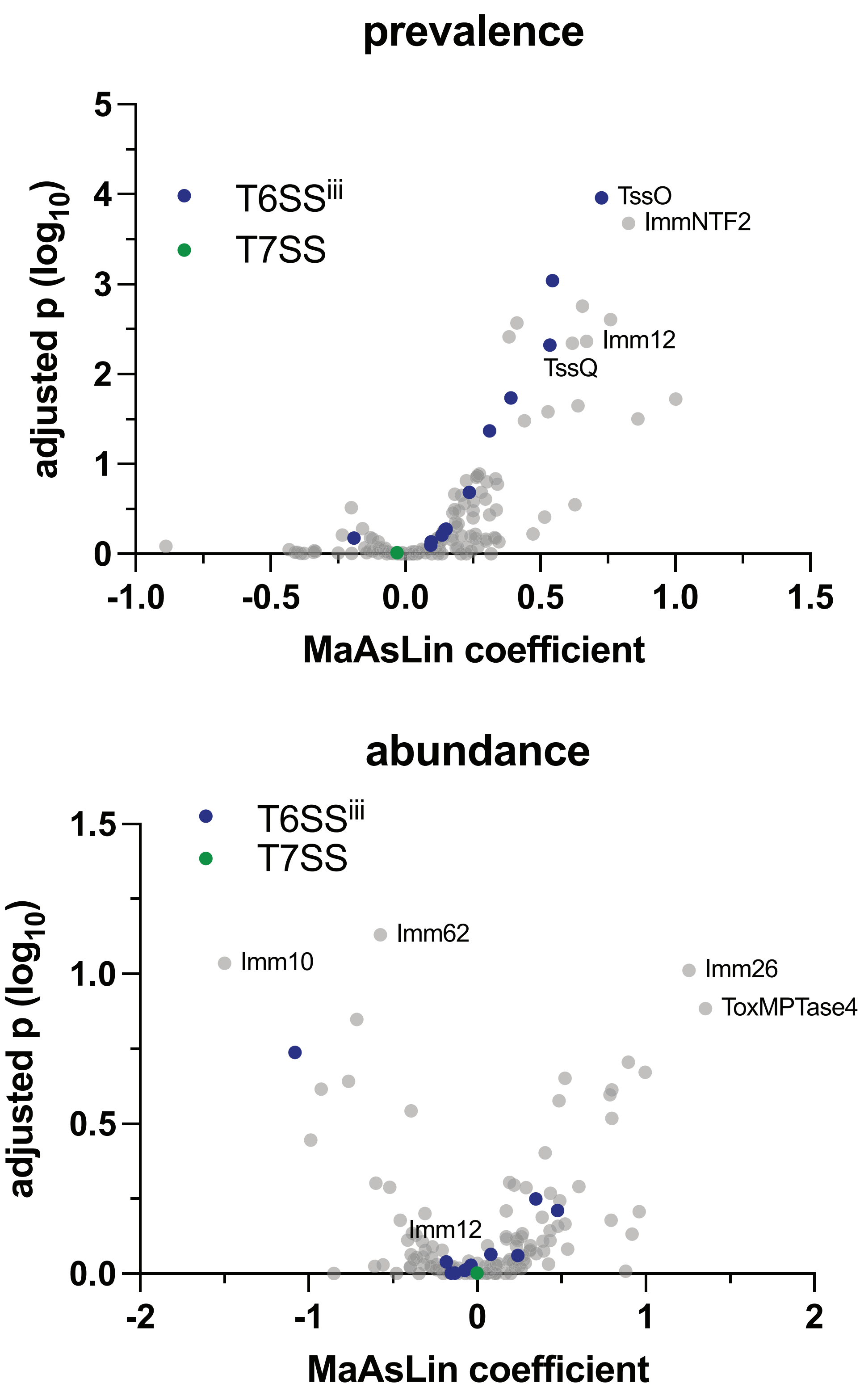

Figure S10. *MaAsLin marker gene analysis by maternal detection and gestational age.* Data corresponding to Figure 4 were assessed with mixed-effects modeling (MaAsLin 3) using numerical gestational age and maternal detection of each marker (present vs. absent) as fixed effects.

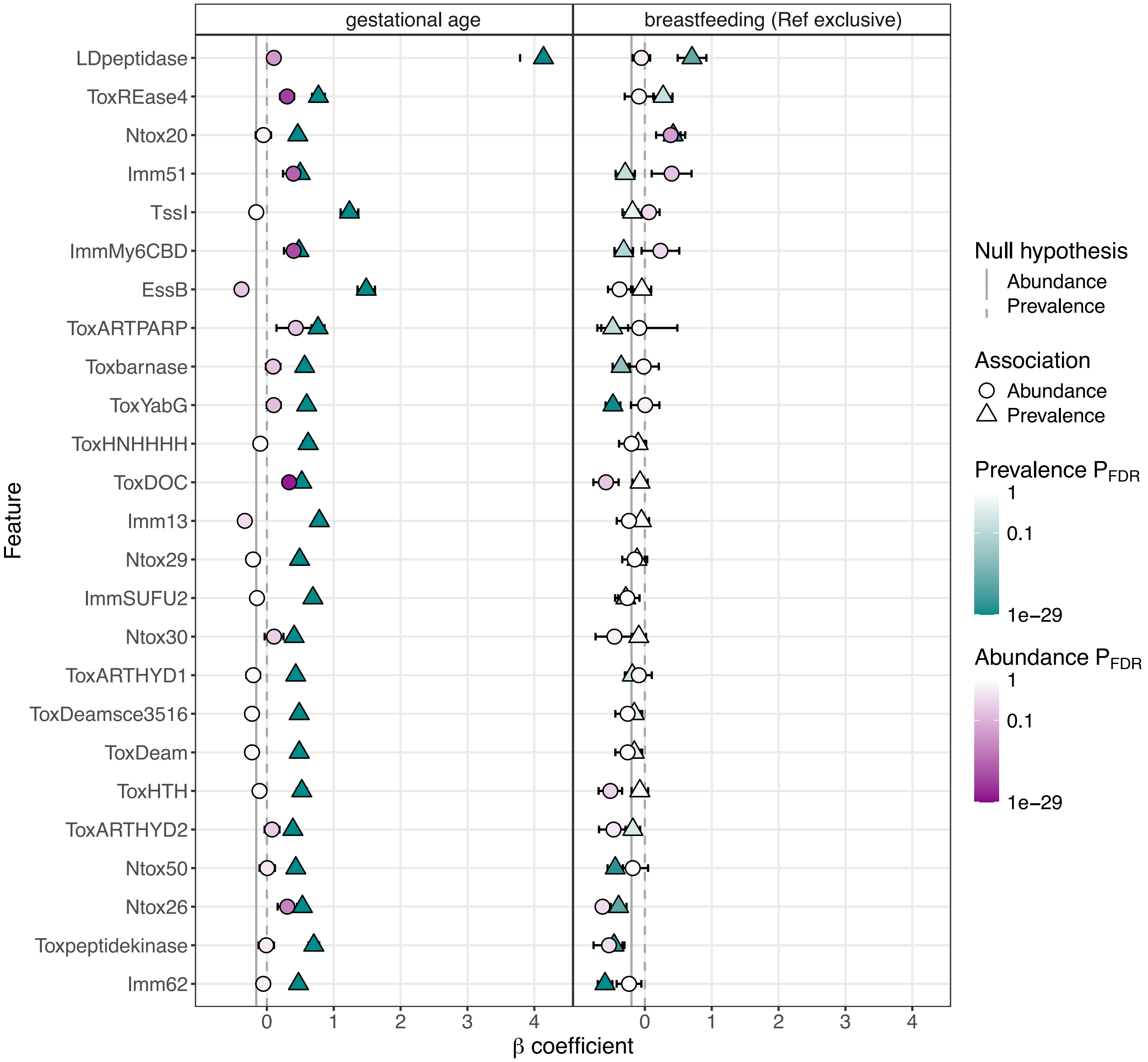

Figure S11. *MaAsLin marker gene analysis by breastfeeding status and gestational age.* Data corresponding to Figure 5 were assessed with mixed-effects modeling (MaAsLin 3) using numerical gestational age and breastfeeding status (exclusive, non-exclusive, unknown) as fixed effects.

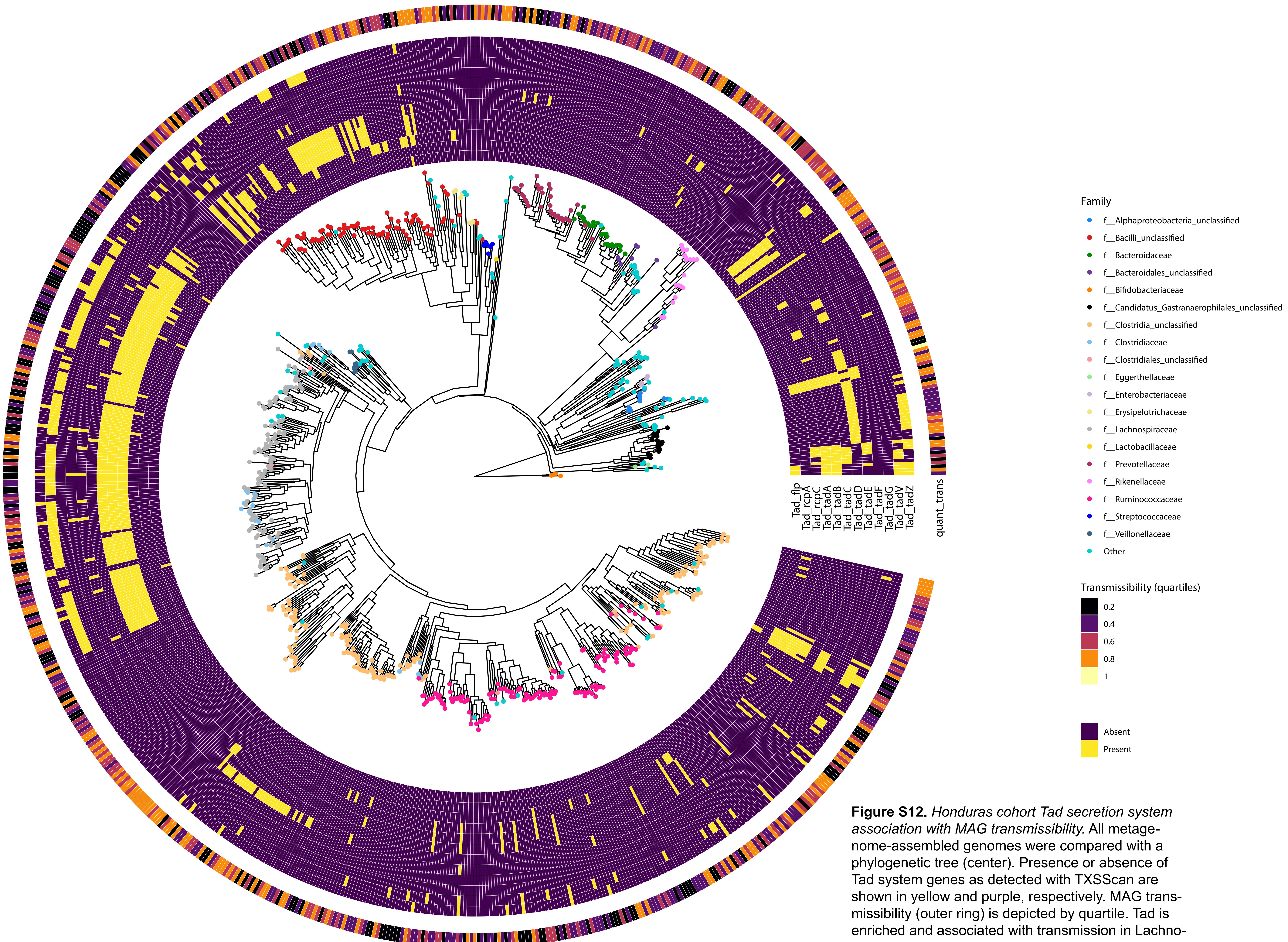

Figure S12. *Honduras cohort Tad secretion system association with MAG transmissibility.* All metagenome-assembled genomes were compared with a phylogenetic tree (center). Presence or absence of Tad system genes as detected with TXSScan are shown in yellow and purple, respectively. MAG transmissibility (outer ring) is depicted by quartile. Tad is enriched and associated with transmission in Lachnospiraceae and Bacilli.

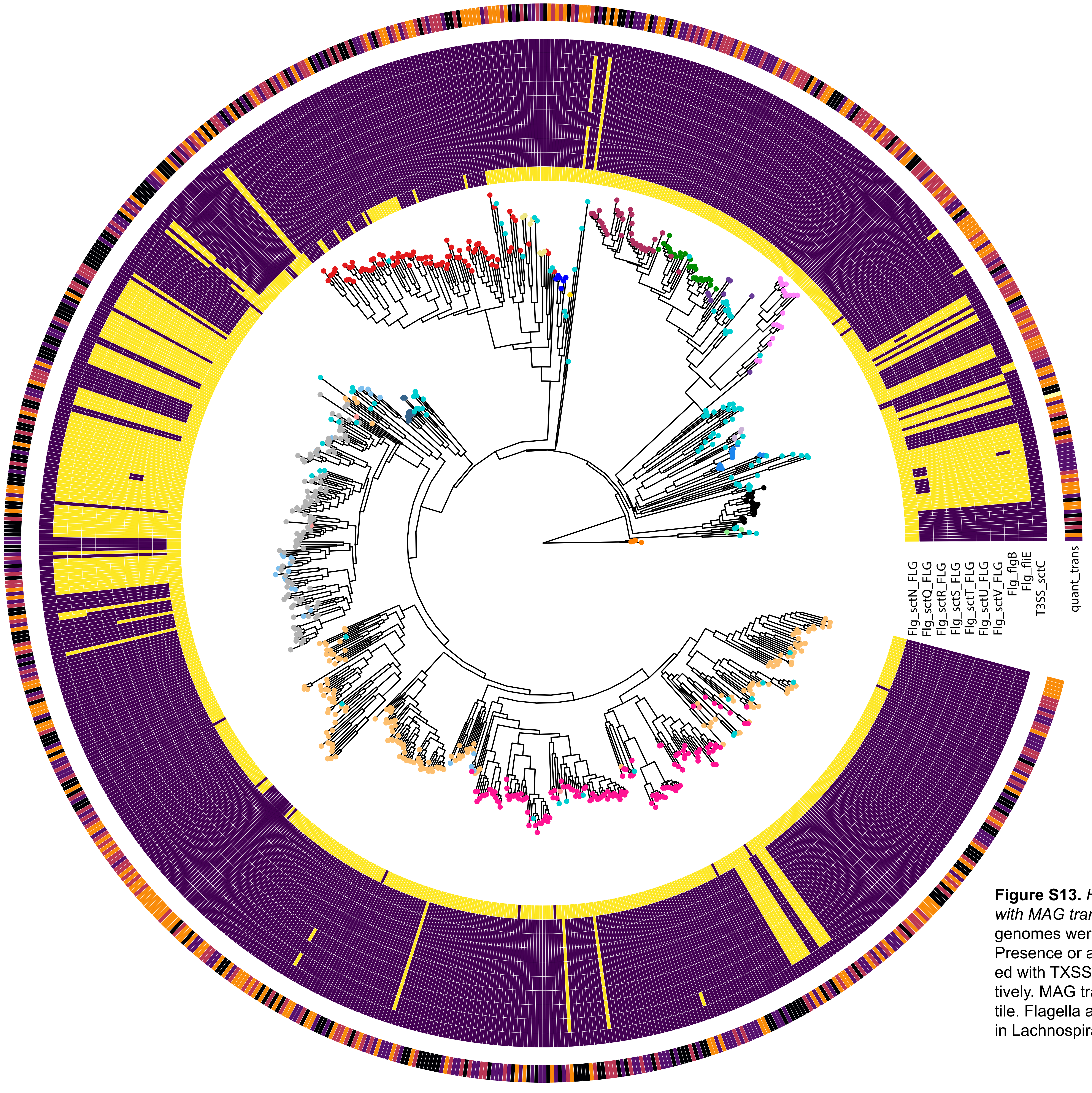

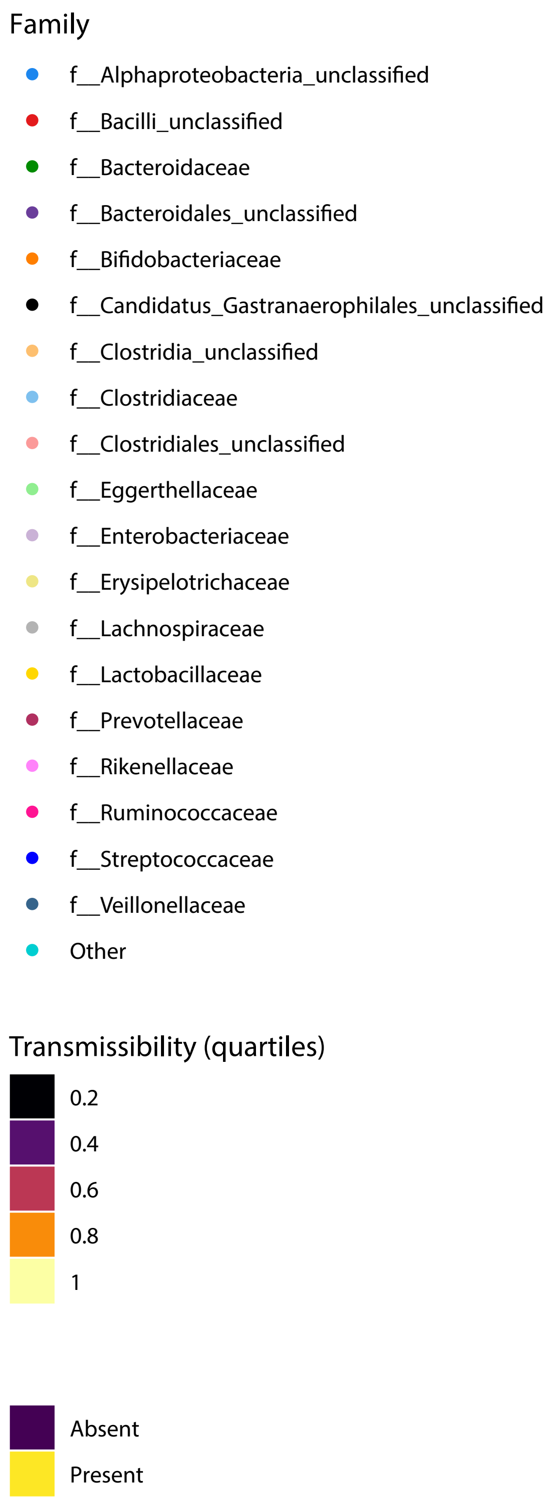

Figure S13. *Honduras cohort flagellum system association with MAG transmissibility.* All metagenome-assembled genomes were compared with a phylogenetic tree (center). Presence or absence of flagellum system genes as detected with TXSScan are shown in yellow and purple, respectively. MAG transmissibility (outer ring) is depicted by quartile. Flagella are enriched and associated with transmission in Lachnospiraceae.

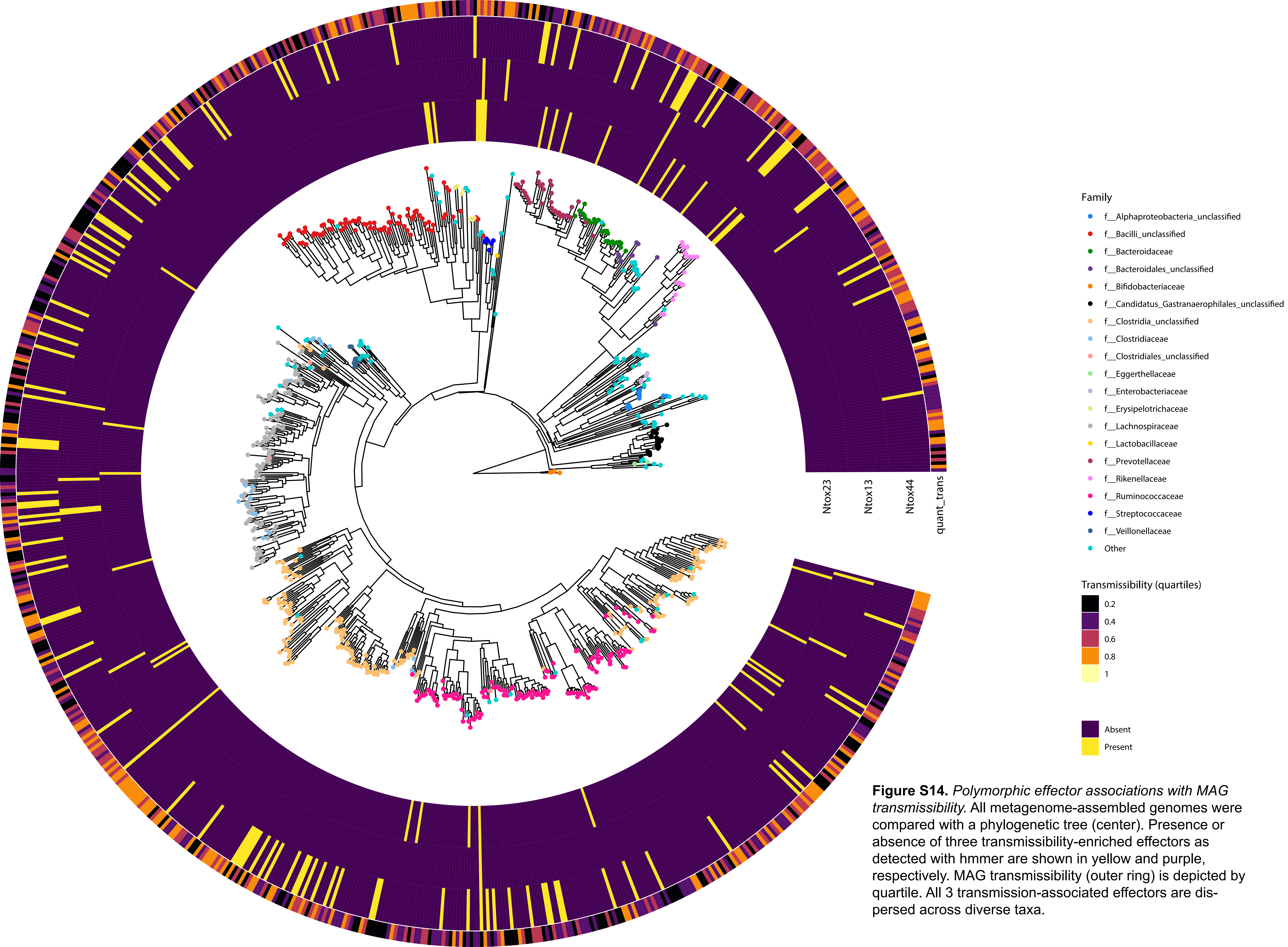

Figure S14. *Polymorphic effector associations with MAG transmissibility.* All metagenome-assembled genomes were compared with a phylogenetic tree (center). Presence or absence of three transmissibility-enriched effectors as detected with hmmer are shown in yellow and purple, respectively. MAG transmissibility (outer ring) is depicted by quartile. All 3 transmission-associated effectors are dispersed across diverse taxa.
