# Supplementary material for: Metagenomic polymorphic toxin effector and immunity profiling predicts microbiome development and disease-related dysbiosis
Source: mSystems. 2026 May 22;11(6):e00305-26. doi: 10.1128/msystems.00305-26 (PMC13288982; doi:10.1128/msystems.00305-26)
Supplement: Supplemental methods — Additional experimental details. [file msystems.00305-26-s0003.pdf]

Supplemental Methods

Data and software resources

Previously published and publicly available shotgun metagenomic data from human fecal specimens were used for PolyProf validation and meta-analysis (Table S2) [1-28]. Taxonomic profiling was performed with Kraken 2 [29]. Polyprof profiling was performed with a custom protein sequence marker database and HUMAnN 3 [30]. The effector/immunity sequence database was constructed using NCBI BLAST [31] and HMMER [32], with the MGnify gut metagenome-assembled genome (MAG) database [33]. Queries were representative sequences and hidden Markov models from each polymorphic toxin effector and immunity class described in the Zhang et al. comprehensive bioinformatic analysis [34]. Also included were Bacteroidales type VI secretion system ($T6SS^{iii}$) conserved structural proteins and the conserved type VII secretion system (T7SS) structural protein EssB. During our database assembly work, we observed frequent association of effector/immunity genes in Bacteroidales MAGs with $T6SS^{iii}$, and we anticipated that $T6SS^{iii}$ genes would be abundant in metagenomes given the dominance of Bacteroidales. The PolyProf database included all sequences of 13 $T6SS^{iii}$ structural genes we identified in MAGs and did not distinguish genetic architectures GA1-3. Because Bacillota are also dominant in microbiomes, and we observed an association of effector/immunity genes with T7SS, we sought to include markers of this system. We observed that Bacillota MAG T7SSb universally encoded *essB* and *essC*, but only variably other structural genes. We constructed marker gene collections for both, but excluded *essC* from PolyProf due to extensive off-target sequence mapping from other ATPase domains (poor specificity). A stringent sequence similarity cutoff was selected to minimize off-target mapping of metagenomic reads (i.e. high specificity). The tradeoff is the potential lack of detection of divergent members of the effector/immunity classes (low sensitivity). Specific sequence inclusion criteria were a BLAST E-value less than 10^{-10} compared to a Zhang et al. curated sequence or an HMMER E-value less than 10^{-10}. Candidate marker sequences were excluded if they contained early stop codons, contained very low

complexity regions, or did not align well with other markers in the class. Within each marker class, Shanon entropies were calculated as a marker of variation among the sequences (Table S1). Enrichment of each effector/immunity marker for each taxonomic lineage was calculated based on the fraction of positive MAGs within the lineage and the corresponding sampling error. Enrichment analyses for all markers are available on the PolyProf GitHub page. Taxonomic trees for each marker were visualized using the R *metacoder* package [35].

The marker sequences were compiled into a custom HUMAnN database using DIAMOND [36]. The marker sequence database is not intended to be exhaustive, as other effector types are known to be secreted through various secretion systems and mediate interbacterial antagonism. However, the PolyProf database used in this study can readily be expanded to include other effector/immunity marker sequences of interest. Other published resources and databases are excellent resources for identifying other effector/immunity classes [37-40]. The PolyProf effector/immunity database and source sequences are publicly available on the PolyProf GitHub page. Abundance tables for all metagenome profiles as part of this study will also be available.

Marker gene performance testing was performed by simulating Illumina-style microbiome data using InSilicoSeq [41]. One million reads were simulated from random selections of 10 known marker-positive MAGs, 100 marker-negative MAGs, or both. Simulation was repeated 10 times for each marker. PolyProf was applied to simulation fastq files, and marker abundance correlated to marker-positive MAG abundance using linear regression with R^2 calculations (GraphPad Prism). 1:1 correlation of these abundances was expected and observed. Deviation of PolyProf marker quantities from the value expected based on MAG abundance was measured for each simulation with mean and 95% confidence interval calculation over the ten replicates to identify markers that were systematically over- or under-estimated by PolyProf. For comparison to an alternative approach, the Zhang et al. HMMs [34] were utilized to detect effector/immunity in translated metagenomic sequences using hmmer

and default parameters. Off-target mapping was quantified as the mean number of hits in 10 marker-negative simulated metagenomes. The limit of detection for each marker was estimated from linear regression models using the International Conference on Harmonization (ICH) method: limit of detection = (3.3 * standard deviation of residuals) / slope [42]. Crossmapping of marker sequences was assessed using CROSSMAPPER [43]. One hundred MAGs negative for each marker sequence were used to generate simulated Metagenomic data, which was then mapped to the PolyProf marker sequence database.

Contributions of metagenome taxonomy to PolyProf variance were estimated with two approaches using the IBD study set. Taxonomic abundances were calculated with MetaPhlAn4 [44]. NMDS analysis (R vegan and phyloseq packages) was performed separately on both the taxa relative abundances and PolyProf, and the dominant NMDS factor values were plotted for each sample. Linear regression was performed and R^2 calculated as an estimate of how much PolyProf variance is explained by taxonomic beta diversity. The second approach to assess each individual marker's relationship to taxonomy was to predict marker abundance from family-level taxonomy and the rate of marker detection in MAGs from each family: *predicted abundance = family detection rate in MAGs * family relative abundance in the metagenome*, summed for all families. This taxonomy-predicted abundance was compared to the PolyProf marker abundance using linear regression and R^2 calculation. Graphs for each marker with linear regression data are available on the PolyProf GitHub site. Contributions of metagenome Bacteroidales abundances to PolyProf associations with diagnoses were assessed using MaAsLin3 multivariable linear modeling of all PolyProf markers by diagnosis, with and without adjustment for Bacteroidales order abundances [45]. Taxonomy contributions to PolyProf in the larger dataset (all diagnoses) were evaluated using partial redundancy analysis (vegan package, R) with PolyProf profiles as the dependent variable, and family-level taxonomy as the independent variable. For performance comparisons, a separate marker protein database was

generated by the same methods, taking effector and immunity sequences from three curated databases: SecReT6, Prokaryotic Antimicrobial Toxin database (PAT), and BastionHub [39,40,46].

Machine learning classifier generation and testing

Binary supervised machine learning models were trained to predict diagnoses with two tiers of polymorphic effector/immunity comparisons: diagnosis *vs*. healthy subjects across all metagenome samples utilized in this study (Table S2), and diagnosis *vs*. all other metagenomes. Each sample consisted of 217 effector or immunity gene target hits from the custom PolyProf HUMAnN database (in reads per kilobase, adjusted for length). For combined taxonomy and PolyProf models, the dataset also included ~5,000 bacterial relative abundances. To prevent data leakage among individual subjects, individuals within longitudinal studies were assigned a host ID and grouped together for model cross-validation, training, and testing. Models were trained using 80% of individuals and tested on a holdout dataset of 20% of individuals. The testing and training datasets were transformed by removing the mean and scaling to a variance of one for each variable, with parameters for scaling determined from the training dataset. Elastic nets were selected for further predictive model development. Parameter tuning was conducted using “IBD” as the diagnosis, which was a combination of “UC” or “CD” diagnoses. Parameter testing was done with a grid of alpha and lambda values. All models in this paper used alpha = 0.5 and lambda = 0.0001. Elastic net models were also constructed using genus-level taxonomy, taxonomy and PolyProf markers, or a collection of curated effector / immunity gene database sequences. The caret package’s train algorithm was implemented in R. Predictive models were evaluated by calculating and plotting the area under the receiver operating curve (AUC) and balanced accuracy. Taxonomy + PolyProf model performance was compared to either taxonomy or PolyProf alone using Friedman tests. Feature stability across folds was quantified as feature selection frequency over 100 folds using the cv.glmnet function in the glmnet R package. Model performance was validated by direct application on external

datasets, not used for training, for 5 diagnoses. Curated metagenomic data and diagnosis metadata were accessed using ExperimentHub's curatedMetagenomicData package [47].

Social strain sharing microbiome analysis

One study [48] included potentially identifying metadata with protected access. For this study, human subjects research was conducted in accordance with the Declaration of Helsinki, with Iowa IRB protocol (#202406297). The original dataset was collected with informed consent of the participants, and this study is a secondary use of the data. PolyProf was applied to metagenome data from a Honduras cohort which had strain sharing scores previously calculated [48]. PolyProf beta diversity was compared by resident village and building identifiers using adonis2 and 2-way ANOVA testing of Bray-Curtis dissimilarities. Pairwise beta diversity distances were correlated with strain sharing rates using linear regression. For each marker, a 2x2 table of strain sharing (zero or not zero) and marker co-detection (present in both subject's metagenomes or not) was constructed. Odds ratios were calculated and p-values derived by Fisher's exact tests.

Metagenome assembled genomes

Metagenomic assembly was performed on all samples using metaSPADES [49] (v. 3.13.1, default parameters), and contigs longer than 1000bp were discarded. Reads were mapped against the assembled contigs using minimap2 [50] (v. 2.24, parameters: -ax sr -t 12 -N50). The resulting alignments were used as input for the VAMB pipeline for contig binning [51] (v. 3.0.9, parameters: --minfasta 500000). The automatic binning procedure generated a total of 226,039 MAGs that were then subjected to quality control to evaluate completeness and contamination using CheckM2 [52] (v. 1.0.2). We selected a total of 130,852 MAGs passing the thresholds for medium-quality genomes, being at least 50% complete and displaying less than 10% of contamination. All MAGs were annotated with Bakta [53] (v. 4.1, default parameters) using

the “Full” database. MAGs were dereplicated at 95% ANI using galah [54], which yielded a total of 2,609 metagenomic species clusters. Cluster representatives were taxonomic annotated with the phylophlan_assign_sgb routine from PhyloPhlAn 3 (v. 3.1.1) [55] using the SGB Jan 21 database. [56]

MAG transmissibility, TXSS, and PolyProf analysis

Previously calculated strain sharing scores were used to calculate species-level transmissibility, which is the number of strain-sharing events detected for a species divided by the total number of potential strain-sharing events for all the species detected by StrainPhlAn4. MAG representatives for each species were used for downstream analysis and transmissibility values were then linked to them. Predicted proteins were used as input for MacSyFinder2 [57] to detect components of secretion systems with the TXSScan [58] using the unordered search mode. In addition to the detection of secretion systems with TXSScan, we aligned the previously built database of effector/immunity sequences against all the predicted proteins using JACKHMMER. The resulting alignment was filtered to retain only domain hits with e-value < 0.001 and domain coverage of at least 50%. Species were ranked according to their transmissibility value and for each effector, enrichment analysis was performed using gene set enrichment analysis as implemented in the fgsea R package [59], by using species with the effector detected as present as gene set. Phylogenetic trees were generated using the Newick tree file provided by MetaPhlAn 4 [44] and annotated with ggtree [60].

References

1. Lloyd-Price, J., Mahurkar, A., Rahnavard, G., Crabtree, J., Orvis, J., Hall, A.B., Brady, A., Creasy, H.H., McCracken, C., Giglio, M.G., et al. (2017). Strains, functions and dynamics in the expanded Human Microbiome Project. Nature *550*, 61–66. 10.1038/nature23889.
2. Raymond, F., Ouameur, A.A., Deraspe, M., Iqbal, N., Gingras, H., Dridi, B., Leprohon, P., Plante, P.L., Giroux, R., Berube, E., et al. (2016). The initial state of the human gut microbiome determines its reshaping by antibiotics. ISME J *10*, 707–720. 10.1038/ismej.2015.148.
3. Fromentin, S., Forslund, S.K., Chechi, K., Aron-Wisnewsky, J., Chakaroun, R., Nielsen, T., Tremaroli, V., Ji, B., Prifti, E., Myridakis, A., et al. (2022). Microbiome and metabolome features of the cardiometabolic disease spectrum. Nat Med *28*, 303–314. 10.1038/s41591-022-01688-4.
4. Monaghan, T.M., Sloan, T.J., Stockdale, S.R., Blanchard, A.M., Emes, R.D., Wilcox, M., Biswas, R., Nashine, R., Manke, S., Gandhi, J., et al. (2020). Metagenomics reveals impact of geography and acute diarrheal disease on the Central Indian human gut microbiome. Gut Microbes *12*, 1752605. 10.1080/19490976.2020.1752605.
5. Kim, J., Cho, Y., Seo, M.R., Bae, M.H., Kim, B., Rho, M., and Pai, H. (2020). Quantitative characterization of Clostridioides difficile population in the gut microbiome of patients with C. difficile infection and their association with clinical factors. Sci Rep *10*, 17608. 10.1038/s41598-020-74090-0.
6. Langdon, A., Schwartz, D.J., Bulow, C., Sun, X., Hink, T., Reske, K.A., Jones, C., Burnham, C.D., Dubberke, E.R., Dantas, G., and Program, C.D.C.P.E. (2021). Microbiota restoration reduces antibiotic-resistant bacteria gut colonization in patients with recurrent Clostridioides difficile infection from the open-label PUNCH CD study. Genome Med *13*, 28. 10.1186/s13073-021-00843-9.
7. Fishbein, S.R., Robinson, J.I., Hink, T., Reske, K.A., Newcomer, E.P., Burnham, C.D., Henderson, J.P., Dubberke, E.R., and Dantas, G. (2022). Multi-omics investigation of Clostridioides difficile-colonized patients reveals pathogen and commensal correlates of C. difficile pathogenesis. Elife *11*. 10.7554/eLife.72801.
8. Verma, S., Dutta, S.K., Firnberg, E., Phillips, L., Vinayek, R., and Nair, P.P. (2021). Identification and engraftment of new bacterial strains by shotgun metagenomic sequence analysis in patients with recurrent Clostridioides difficile infection before and after fecal microbiota transplantation and in healthy human subjects. PLoS One *16*, e0251590. 10.1371/journal.pone.0251590.
9. Ma, Y., Zhang, Y., Xiang, J., Xiang, S., Zhao, Y., Xiao, M., Du, F., Ji, H., Kaboli, P.J., Wu, X., et al. (2021). Metagenome Analysis of Intestinal Bacteria in Healthy People, Patients With Inflammatory Bowel Disease and Colorectal Cancer. Front Cell Infect Microbiol *11*, 599734. 10.3389/fcimb.2021.599734.
10. Vogtmann, E., Hua, X., Zeller, G., Sunagawa, S., Voigt, A.Y., Hercog, R., Goedert, J.J., Shi, J., Bork, P., and Sinha, R. (2016). Colorectal Cancer and the Human Gut Microbiome: Reproducibility with Whole-Genome Shotgun Sequencing. PLoS One *11*, e0155362. 10.1371/journal.pone.0155362.
11. Wu, Y., Jiao, N., Zhu, R., Zhang, Y., Wu, D., Wang, A.J., Fang, S., Tao, L., Li, Y., Cheng, S., et al. (2021). Identification of microbial markers across populations in early detection of colorectal cancer. Nat Commun *12*, 3063. 10.1038/s41467-021-23265-y.
12. Liu, N.N., Jiao, N., Tan, J.C., Wang, Z., Wu, D., Wang, A.J., Chen, J., Tao, L., Zhou, C., Fang, W., et al. (2022). Multi-kingdom microbiota analyses identify bacterial-fungal

interactions and biomarkers of colorectal cancer across cohorts. Nat Microbiol *7*, 238–250. 10.1038/s41564-021-01030-7.
13. Xu, J., Zheng, Z., Yang, L., Li, R., Ma, X., Zhang, J., Yin, F., Liu, L., Xu, Q., Shen, Q., et al. (2022). A novel promising diagnosis model for colorectal advanced adenoma and carcinoma based on the progressive gut microbiota gene biomarkers. Cell Biosci *12*, 208. 10.1186/s13578-022-00940-1.
14. Lewis, J.D., Chen, E.Z., Baldassano, R.N., Otley, A.R., Griffiths, A.M., Lee, D., Bittinger, K., Bailey, A., Friedman, E.S., Hoffmann, C., et al. (2015). Inflammation, Antibiotics, and Diet as Environmental Stressors of the Gut Microbiome in Pediatric Crohn's Disease. Cell Host Microbe *18*, 489–500. 10.1016/j.chom.2015.09.008.
15. Lee, J.W.J., Plichta, D., Hogstrom, L., Borren, N.Z., Lau, H., Gregory, S.M., Tan, W., Khalili, H., Clish, C., Vlamakis, H., et al. (2021). Multi-omics reveal microbial determinants impacting responses to biologic therapies in inflammatory bowel disease. Cell Host Microbe *29*, 1294–1304 e1294. 10.1016/j.chom.2021.06.019.
16. Lloyd-Price, J., Arze, C., Ananthakrishnan, A.N., Schirmer, M., Avila-Pacheco, J., Poon, T.W., Andrews, E., Ajami, N.J., Bonham, K.S., Brislawn, C.J., et al. (2019). Multi-omics of the gut microbial ecosystem in inflammatory bowel diseases. Nature *569*, 655–662. 10.1038/s41586-019-1237-9.
17. Johnson, K.E., Hernandez-Alvarado, N., Blackstad, M., Heisel, T., Allert, M., Fields, D.A., Isganaitis, E., Jacobs, K.M., Knights, D., Lock, E.F., et al. (2024). Human cytomegalovirus in breast milk is associated with milk composition and the infant gut microbiome and growth. Nat Commun *15*, 6216. 10.1038/s41467-024-50282-4.
18. Korpela, K., Costea, P., Coelho, L.P., Kandels-Lewis, S., Willemsen, G., Boomsma, D.I., Segata, N., and Bork, P. (2018). Selective maternal seeding and environment shape the human gut microbiome. Genome Res *28*, 561–568. 10.1101/gr.233940.117.
19. Shao, Y., Forster, S.C., Tsaliki, E., Vervier, K., Strang, A., Simpson, N., Kumar, N., Stares, M.D., Rodger, A., Brocklehurst, P., et al. (2019). Stunted microbiota and opportunistic pathogen colonization in caesarean-section birth. Nature *574*, 117–121. 10.1038/s41586-019-1560-1.
20. Backhed, F., Roswall, J., Peng, Y., Feng, Q., Jia, H., Kovatcheva-Datchary, P., Li, Y., Xia, Y., Xie, H., Zhong, H., et al. (2015). Dynamics and Stabilization of the Human Gut Microbiome during the First Year of Life. Cell Host Microbe *17*, 690–703. 10.1016/j.chom.2015.04.004.
21. Aguilar-Lopez, M., Wetzel, C., MacDonald, A., Ho, T.T.B., and Donovan, S.M. (2022). Metagenomic profile of the fecal microbiome of preterm infants consuming mother's own milk with bovine milk-based fortifier or infant formula: a cross-sectional study. Am J Clin Nutr *116*, 435–445. 10.1093/ajcn/nqac081.
22. Chen, J., Wang, A., and Wang, Q. (2021). Dysbiosis of the gut microbiome is a risk factor for osteoarthritis in older female adults: a case control study. BMC Bioinformatics *22*, 299. 10.1186/s12859-021-04199-0.
23. Tagliamonte, S., Laiola, M., Ferracane, R., Vitale, M., Gallo, M.A., Meslier, V., Pons, N., Ercolini, D., and Vitaglione, P. (2021). Mediterranean diet consumption affects the endocannabinoid system in overweight and obese subjects: possible links with gut microbiome, insulin resistance and inflammation. Eur J Nutr *60*, 3703–3716. 10.1007/s00394-021-02538-8.
24. Guo, R., Li, S., Zhang, Y., Zhang, Y., Wang, G., Ullah, H., Ma, Y., and Yan, Q. (2022). Dysbiotic Oral and Gut Viromes in Untreated and Treated Rheumatoid Arthritis Patients. Microbiol Spectr *10*, e0034822. 10.1128/spectrum.00348-22.
25. Fluhr, L., Mor, U., Kolodziejczyk, A.A., Dori-Bachash, M., Leshem, A., Itav, S., Cohen, Y., Suez, J., Zmora, N., Moresi, C., et al. (2021). Gut microbiota modulates weight gain in

mice after discontinued smoke exposure. Nature *600*, 713–719. 10.1038/s41586-021-04194-8.

26. Zhao, L., Zhang, F., Ding, X., Wu, G., Lam, Y.Y., Wang, X., Fu, H., Xue, X., Lu, C., Ma, J., et al. (2018). Gut bacteria selectively promoted by dietary fibers alleviate type 2 diabetes. Science *359*, 1151–1156. 10.1126/science.aao5774.
27. Qin, J., Li, Y., Cai, Z., Li, S., Zhu, J., Zhang, F., Liang, S., Zhang, W., Guan, Y., Shen, D., et al. (2012). A metagenome-wide association study of gut microbiota in type 2 diabetes. Nature *490*, 55–60. 10.1038/nature11450.
28. Bacorn, M., Subramanian, P., Levy, S., Chen, Q., Maxwell, G.L., and Hourigan, S.K. (2024). Faecal zonulin, calprotectin and the infant microbiome in early life. Clin Transl Med *14*, e1695. 10.1002/ctm2.1695.
29. Lu, J., Rincon, N., Wood, D.E., Breitwieser, F.P., Pockrandt, C., Langmead, B., Salzberg, S.L., and Steinegger, M. (2022). Metagenome analysis using the Kraken software suite. Nat Protoc *17*, 2815–2839. 10.1038/s41596-022-00738-y.
30. Beghini, F., McIver, L.J., Blanco-Miguez, A., Dubois, L., Asnicar, F., Maharjan, S., Mailyan, A., Manghi, P., Scholz, M., Thomas, A.M., et al. (2021). Integrating taxonomic, functional, and strain-level profiling of diverse microbial communities with bioBakery 3. Elife *10*. 10.7554/eLife.65088.
31. Camacho, C., Coulouris, G., Avagyan, V., Ma, N., Papadopoulos, J., Bealer, K., and Madden, T.L. (2009). BLAST+: architecture and applications. BMC Bioinformatics *10*, 421. 10.1186/1471-2105-10-421.
32. Potter, S.C., Luciani, A., Eddy, S.R., Park, Y., Lopez, R., and Finn, R.D. (2018). HMMER web server: 2018 update. Nucleic Acids Res *46*, W200–W204. 10.1093/nar/gky448.
33. Richardson, L., Allen, B., Baldi, G., Beracochea, M., Bileschi, M.L., Burdett, T., Burgin, J., Caballero-Perez, J., Cochrane, G., Colwell, L.J., et al. (2023). MGnify: the microbiome sequence data analysis resource in 2023. Nucleic Acids Res *51*, D753–D759. 10.1093/nar/gkac1080.
34. Zhang, D., de Souza, R.F., Anantharaman, V., Iyer, L.M., and Aravind, L. (2012). Polymorphic toxin systems: Comprehensive characterization of trafficking modes, processing, mechanisms of action, immunity and ecology using comparative genomics. Biol Direct *7*, 18. 10.1186/1745-6150-7-18.
35. Rice, P., Longden, I., and Bleasby, A. (2000). EMBOSS: the European Molecular Biology Open Software Suite. Trends Genet *16*, 276–277. 10.1016/s0168-9525(00)02024-2.
36. Buchfink, B., Reuter, K., and Drost, H.G. (2021). Sensitive protein alignments at tree-of-life scale using DIAMOND. Nat Methods *18*, 366–368. 10.1038/s41592-021-01101-x.
37. Li, J., Yao, Y., Xu, H.H., Hao, L., Deng, Z., Rajakumar, K., and Ou, H.Y. (2015). SecReT6: a web-based resource for type VI secretion systems found in bacteria. Environ Microbiol *17*, 2196–2202. 10.1111/1462-2920.12794.
38. Danov, A., Segev, O., Bograd, A., Ben Eliyahu, Y., Dotan, N., Kaplan, T., and Levy, A. (2024). Toxinome-the bacterial protein toxin database. mBio *15*, e0191123. 10.1128/mbio.01911-23.
39. Liu, Y., Liu, S., Pan, Z., Ren, Y., Jiang, Y., Wang, F., Li, D.D., Li, Y.Z., and Zhang, Z. (2023). PAT: a comprehensive database of prokaryotic antimicrobial toxins. Nucleic Acids Res *51*, D452–D459. 10.1093/nar/gkac879.
40. Wang, J., Li, J., Hou, Y., Dai, W., Xie, R., Marquez-Lago, T.T., Leier, A., Zhou, T., Torres, V., Hay, I., et al. (2021). BastionHub: a universal platform for integrating and analyzing substrates secreted by Gram-negative bacteria. Nucleic Acids Res *49*, D651–D659. 10.1093/nar/gkaa899.
41. Gourle, H., Karlsson-Lindsjo, O., Hayer, J., and Bongcam-Rudloff, E. (2019). Simulating Illumina metagenomic data with InSilicoSeq. Bioinformatics *35*, 521–522. 10.1093/bioinformatics/bty630.

42. International Conference on Harmonisation of technical requirements for registration of pharmaceuticals for human, u. (2001). ICH harmonized tripartite guideline: Guideline for Good Clinical Practice. J Postgrad Med *47*, 45–50.
43. Hovhannisyan, H., Hafez, A., Llorens, C., and Gabaldon, T. (2020). CROSSMAPPER: estimating cross-mapping rates and optimizing experimental design in multi-species sequencing studies. Bioinformatics *36*, 925–927. 10.1093/bioinformatics/btz626.
44. Blanco-Miguez, A., Beghini, F., Cumbo, F., McIver, L.J., Thompson, K.N., Zolfo, M., Manghi, P., Dubois, L., Huang, K.D., Thomas, A.M., et al. (2023). Extending and improving metagenomic taxonomic profiling with uncharacterized species using MetaPhlAn 4. Nat Biotechnol *41*, 1633–1644. 10.1038/s41587-023-01688-w.
45. Nickols, W.A., Kuntz, T., Shen, J., Maharjan, S., Mallick, H., Franzosa, E.A., Thompson, K.N., Nearing, J.T., and Huttenhower, C. (2024). MaAsLin 3: Refining and extending generalized multivariable linear models for meta-omic association discovery. bioRxiv. 10.1101/2024.12.13.628459.
46. Zhang, J., Guan, J., Wang, M., Li, G., Djordjevic, M., Tai, C., Wang, H., Deng, Z., Chen, Z., and Ou, H.Y. (2023). SecReT6 update: a comprehensive resource of bacterial Type VI Secretion Systems. Sci China Life Sci *66*, 626–634. 10.1007/s11427-022-2172-x.
47. Pasolli, E., Schiffer, L., Manghi, P., Renson, A., Obenchain, V., Truong, D.T., Beghini, F., Malik, F., Ramos, M., Dowd, J.B., et al. (2017). Accessible, curated metagenomic data through ExperimentHub. Nat Methods *14*, 1023–1024. 10.1038/nmeth.4468.
48. Beghini, F., Pullman, J., Alexander, M., Shridhar, S.V., Prinster, D., Singh, A., Matute Juarez, R., Airoldi, E.M., Brito, I.L., and Christakis, N.A. (2024). Gut microbiome strain-sharing within isolated village social networks. Nature. 10.1038/s41586-024-08222-1.
49. Nurk, S., Meleshko, D., Korobeynikov, A., and Pevzner, P.A. (2017). metaSPAdes: a new versatile metagenomic assembler. Genome Res. *27*, 824–834. 10.1101/gr.213959.116.
50. Li, H. (2018). Minimap2: pairwise alignment for nucleotide sequences. Bioinformatics *34*, 3094–3100. 10.1093/bioinformatics/bty191.
51. Nissen, J.N., Johansen, J., Allesøe, R.L., Sønderby, C.K., Armenteros, J.J.A., Grønbech, C.H., Jensen, L.J., Nielsen, H.B., Petersen, T.N., Winther, O., and Rasmussen, S. (2021). Improved metagenome binning and assembly using deep variational autoencoders. Nat. Biotechnol. *39*, 555–560. 10.1038/s41587-020-00777-4.
52. Chklovski, A., Parks, D.H., Woodcroft, B.J., and Tyson, G.W. (2023). CheckM2: a rapid, scalable and accurate tool for assessing microbial genome quality using machine learning. Nat. Methods *20*, 1203–1212. 10.1038/s41592-023-01940-w.
53. Schwengers, O., Jelonek, L., Dieckmann, M.A., Beyvers, S., Blom, J., and Goesmann, A. (2021). Bakta: rapid and standardized annotation of bacterial genomes via alignment-free sequence identification. Microb. Genom. *7*. 10.1099/mgen.0.000685.
54. Woodcroft, B.J. (2024). galah: More scalable dereplication for metagenome assembled genomes.
55. Asnicar, F., Thomas, A.M., Beghini, F., Mengoni, C., Manara, S., Manghi, P., Zhu, Q., Bolzan, M., Cumbo, F., May, U., et al. (2020). Precise phylogenetic analysis of microbial isolates and genomes from metagenomes using PhyloPhlAn 3.0. Nat. Commun. *11*, 2500. 10.1038/s41467-020-16366-7.
56. Chaumeil, P.-A., Mussig, A.J., Hugenholtz, P., and Parks, D.H. (2022). GTDB-Tk v2: memory friendly classification with the genome taxonomy database. Bioinformatics *38*, 5315–5316. 10.1093/bioinformatics/btac672.
57. Néron, B., Denise, R., Coluzzi, C., Touchon, M., Rocha, E.P.C., and Abby, S.S. (2023). MacSyFinder v2: Improved modelling and search engine to identify molecular systems in genomes. Peer Community Journal *3*. 10.24072/pcjournal.250.

58. Abby, S.S., Denise, R., and Rocha, E.P.C. (2024). Identification of Protein Secretion Systems in Bacterial Genomes Using MacSyFinder Version 2. Methods Mol Biol *2715*, 1–25. 10.1007/978-1-0716-3445-5_1.
59. Korotkevich, G., Sukhov, V., Budin, N., Shpak, B., Artyomov, M.N., and Sergushichev, A. (2021). 10.1101/060012.
60. Xu, S., Li, L., Luo, X., Chen, M., Tang, W., Zhan, L., Dai, Z., Lam, T.T., Guan, Y., and Yu, G. (2022). Ggtree: A serialized data object for visualization of a phylogenetic tree and annotation data. Imeta *1*, e56. 10.1002/imt2.56.
